# Differential abundance of lipids and metabolites related to SARS-CoV-2 infection and susceptibility

**DOI:** 10.1038/s41598-023-40999-5

**Published:** 2023-09-13

**Authors:** Oihane E. Albóniga, Elena Moreno, Javier Martínez-Sanz, Pilar Vizcarra, Raquel Ron, Jorge Díaz-Álvarez, Marta Rosas Cancio-Suarez, Matilde Sánchez-Conde, Juan Carlos Galán, Santiago Angulo, Santiago Moreno, Coral Barbas, Sergio Serrano-Villar

**Affiliations:** 1 Centro de Metabolómica y Bioanálisis (CEMBIO), Facultad de Farmacia, Universidad San Pablo-CEU, CEU Universities, Urbanización Montepríncipe, Boadilla del Monte, 28660 Madrid, Spain; 2 Department of Infectious Diseases, Hospital Universitario Ramón y Cajal, IRYCIS, 28034 Madrid, Spain; 3 CIBERINFEC, Instituto de Salud Carlos III, Madrid, Spain; 4 Department of Microbiology, Hospital Universitario Ramón y Cajal, IRYCIS, 28034 Madrid, Spain; 5 CIBERESP, Instituto de Salud Carlos III, Madrid, Spain; 6 Department of Infectious Diseases, Hospital Universitario Ramon y Cajal, Facultad de Medicina, Universidad de Alcalá (IRYCIS), Carretera de Colmenar Viejo, Km 9.100, 28034 Madrid, Spain

**Keywords:** SARS-CoV-2, Biomarkers, Molecular medicine, Metabolomics

## Abstract

The mechanisms driving SARS-CoV-2 susceptibility remain poorly understood, especially the factors determining why unvaccinated individuals remain uninfected despite high-risk exposures. To understand lipid and metabolite profiles related with COVID-19 susceptibility and disease progression. We collected samples from an exceptional group of unvaccinated healthcare workers heavily exposed to SARS-CoV-2 but not infected (‘non-susceptible’) and subjects who became infected during the follow-up (‘susceptible’), including non-hospitalized and hospitalized patients with different disease severity providing samples at early disease stages. Then, we analyzed their plasma metabolomic profiles using mass spectrometry coupled with liquid and gas chromatography. We show specific lipids profiles and metabolites that could explain SARS-CoV-2 susceptibility and COVID-19 severity. More importantly, non-susceptible individuals show a unique lipidomic pattern characterized by the upregulation of most lipids, especially ceramides and sphingomyelin, which could be interpreted as markers of low susceptibility to SARS-CoV-2 infection. This study strengthens the findings of other researchers about the importance of studying lipid profiles as relevant markers of SARS-CoV-2 pathogenesis.

## Introduction

Why some unvaccinated individuals with repeated high-risk exposures to SARS-CoV-2 did not show evidence of COVID-19 during the first pandemic waves? Among the possible explanations, host factors have been shown drive SARS-CoV-2 susceptibility and COVID-19 disease severity^1^. Consequently, omics studies, including metabolomic profiling, have gained attention to elucidate biochemical pathways affected by SARS-CoV-2 infection^2–4^. Metabolic profiles can be obtained by mass spectrometry (MS) coupled with different separation techniques, such as liquid chromatography (LC–MS), gas chromatography (GC–MS), or capillary electrophoresis (CE–MS). These techniques allow the identification of different types of molecules, such as amino acids, lipids, and glycoproteins^5^. Since it is not possible to analyze the vast amount of metabolites present in plasma samples using only one technique, the combination of these complementary techniques provides a broader picture of the metabolic pathways and their metabolites under selected conditions.

The COVID-19 pandemic has generated a considerable economic and societal impact^6^ including long-term effects^7,8^ due to Long COVID conditions^9^. Abnormal immune responses to SARS-CoV-2 characterized by impaired macrophage, neutrophil, and dendritic functions or decreased IFN-ɣ production appear to explain adverse clinical outcomes^10–12^. Furthermore, several comorbidities such as immunosuppression, diabetes, pulmonary disease, or cardiovascular disease negatively affect disease progression^13,14^. Lipidomics and metabolomics have been used to predict severity of disease and identify prognostic markers^15–21^. However, the underlying mechanisms of these characteristics of COVID-19 disease remain unclear. Long COVID individuals experience persistent symptoms and complications beyond the acute phase with symptoms such as fatigue, shortness of breath, cognitive difficulties, etc. (reviewed by Davis et al.^22^) Furthermore, COVID-19 symptoms in general can vary widely among individuals, affecting not only the respiratory system but also to other organ systems, leading to gastrointestinal, cardiovascular, neurological and dermatological manifestations. The disease progression and severity might vary also between individuals, especially those with underlying conditions, compromised immune systems, elderly, etc^23^.

To address this question, we performed this exploratory study during the first wave in Madrid in unvaccinated individuals. Importantly, we included healthcare workers involved in COVID-19 care that did not show any clinical or serologic evidence of developing infection despite repeated high-risk exposures to SARS-CoV-2, and we compared them with non-hospitalized and hospitalized patients with different COVID-19 severity at early stages of the disease. We analyzed by different MS techniques the most relevant lipids and metabolites related to SARS-CoV-2 susceptibility. Here, we complete the profile of the metabolites previously reported using CE-MS^24^ with the metabolites obtained in this work by GC–MS and lipids by LC–MS to provide a more complete idea of the molecular principles influencing SARS-CoV-2 susceptibility.

## Methods

### Reagents

All reagents, solvents, and standards used for sample treatment and subsequent analysis are described in the Supporting Information.

### Study design and setting

Study participants were classified according to the presence of SARS-CoV-2 infection at the time of study sampling, and their susceptibility to SARS-CoV-2. Participants with COVID-19 (COVID-19^+^ group) included hospitalized patients with confirmed SARS-CoV-2 infection by polymerase chain reaction (PCR) from nasopharyngeal swabs, sputum, or lower respiratory tract secretions within the first 7 days from the onset of symptoms, who were then classified according to clinical severity as a mild disease, defined as those without a need for supplemental oxygen and who were asymptomatic 1 week after diagnosis; moderate disease, defined as the presence of bilateral radiologic infiltrates or opacities and clinical assessment requiring supplemental oxygen; and severe disease, defined as the development of acute respiratory distress syndrome^25^, Participants without COVID-19 (COVID-19^−^) were asymptomatic healthcare workers with a negative PCR against SARS-CoV-2 in nasopharyngeal exudate.

To assess the differences according to the susceptibility to SARS-CoV-2 infection, we sought to avoid the potential confounding effect of active COVID-19 on the plasma metabolome, so we restricted the analyses to the healthcare workers with confirmed previous COVID-19 (‘susceptible’), by a positive IgG for SARS-CoV-2 or previous COVID-19 confirmed by PCR from nasopharyngeal exudate but not actively ill during recruitment. ‘Non-susceptible’ participants were healthy healthcare workers who were on duty for at least three months in COVID-19 wards or intensive care units and reported at least three high-risk exposures to SARS-CoV-2^24,26^ without having experienced symptoms suggestive of SARS-CoV-2 infection, were persistently negative for SARS-CoV-2 PCR testing and did not have SARS-CoV-2 IgM and IgG in plasma. The most frequent exposure was largely unprotected exposure to aerosol-generating procedures or patient secretions and close contact without facemasks with other confirmed cases of COVID-19.

We measured SARS-CoV-2 antibodies by indirect chemiluminescence immunoassay (Vircell, Granada, Spain).

### Sample preparation

Cryopreserved plasma was treated for safety with previous sample manipulation with inactivation of the SARS-CoV-2 virus and deproteinization by adding 1500 µL of cold methanol:ethanol (MeOH:EtOH) in 1:1 (v/v) proportion to 500 µL of plasma. Further steps to perform LC–MS and GC–MS are detailed in the Supporting Information Methods section. For GC–MS, a Gerstel Multiple Purpose Sample (MPS) Preparation Station (Gerstel MPS PrepStation; GERSTEL, Inc, Maryland, USA) was used for sample derivatization (see Supporting Information for more details).

### GC–MS and LC–MS metabolic fingerprinting

To increase the metabolite coverage, the extracted plasma samples were analyzed by two platforms commonly employed in metabolomics studies: LC–MS, liquid chromatography-mass spectrometry, and GC–MS, gas chromatography-mass spectrometry. For LC–MS, plasma metabolome was acquired using an Agilent 1290 Infinity II UHPLC system coupled to an Agilent 6545 quadrupole time-of-flight (QTOF) mass spectrometer, equipped with an electrospray ionization source (ESI), and operating in positive and negative ionization modes. Samples were analyzed in MS and at the end of the run sequence a QC sample was analyzed in iterative MS/MS following Agilent’s protocol^27^. For GC–MS, samples were analyzed in an Agilent GC–MS system (8890) coupled to a single quadrupole mass spectrometer (5977B, Agilent Technologies). The analysis in LC–MS and GC–MS was performed using previously reported methods with the analytical conditions described in detail in the Supporting Information. In both, LC–MS and GC–MS analysis, the analytical sequence was as follows: QCs were analyzed throughout the sequence, at the beginning and the end for system conditioning, as well as along the sequence for system reproducibility every 8 samples. A pair of blanks were injected at the beginning before QCs and at the end of the run to remove metabolites coming from the sample preparation or mobile phase solvents. Samples were randomly analyzed through the sequence in order to reduce any time-related effect.

### Data treatment

The acquired data in positive and negative ionization modes obtained by LC–MS, as well as the GC–MS data followed a specific analysis pattern further described in the Supporting Information section “Data Processing”.

### Statistical analysis

For the comparison of the general characteristics, we used the Chi^2^ test for categorical variables and the Wilcoxon rank test for continuous variables. Two different comparisons were performed to evaluate COVID-19 disease and susceptibility. The number of samples of mild, moderate, and severe cases were insufficient for powered comparisons, but we explored the differences to inform further studies focused on severity. Subjects were classified as COVID-19+ or COVID-19− according to disease diagnosis; susceptible or non-susceptible according to disease susceptibility; and mild, moderate, or severe according to disease severity. The filtered matrix obtained in the previous step was processed by SIMCA-P version 16.0.1 (Umetrics, Umea, Sweden), MATLAB software (R2018b, The MathWorks, Maticks, MA, USA), MetaboAnalyst 5.0, and SPSS version 27 (IBM SPSS Statistics). When needed, the intensity drop was corrected with the intra-batch effect correction using quality control samples and support vector regression (QC-SVRC)^28^. All the statistical processes are described in detail in the Supporting Information.

### Metabolite identification

The annotation process in GC–MS was carried out during the data treatment by using several libraries such as the Fiehn Library (version 2008), an ‘in-house’ plasma library, and the National Institute of Standards and Technology (NIST) library 2.2 (version 2014). Furthermore, those unknown compounds that were found to be statistically significant were further investigated with the MS Interpreter, incorporated in the NIST MS Search version 2.3, and ChemSketch MS Fragmenter (ACD/Labs, version 2018.2.1) for production and fragmentation structure prediction. Specific details for each technique are detailed in the Supporting Information file.

For LC–MS, accurate m/z was compared with putative metabolites with a mass tolerance of 5 ppm. Then, fragments obtained experimentally were compared with the libraries incorporated in MS-Dial, LipidAnnotator and CEU Mass Mediator^29–31^.

Compilation of the data obtained by the two MS techniques in Table S1 shows matrices of 588 and 686 lipids for LC–MS positive and negative ionization modes, respectively, and of 115 metabolites for GC–MS.

It is important to point out that, consistently with our previous study^24^ any metabolite associated with COVID-19 treatments or drugs that were identified among the significant metabolites were excluded from both MVDA and UVDA statistical analysis. Moreover, among the list of drugs provided by the clinicians, only hydroxychloroquine was found in the LC–MS matrix at positive ionization mode (m/z 336.1838—RT 0.82 min). Finally, the Lipid Network Explorer (LINEX, version 1)^32^ was used for lipid networking to see which ones were connected and the reaction type between them.

### Study approval

The study was carried out at the Ramón and Cajal University Hospital in Madrid (Spain) and was approved by the local research ethics committee (Comite etico de Investigacion clinica GAE Ramon y Cajal, ceic.hrc@salud.madrid.org, approval number 095/20). Informed consent was obtained from all subjects and/or their legal guardian(s). All methods were performed in accordance with the relevant guidelines and regulations.

## Results

### General characteristics of the study population

We included a total of 63 adults that were classified as COVID-19+ (n = 27) and COVID-19− (n = 36) for disease status, and as susceptible (n = 12) and non-susceptible (n = 26) for susceptibility, considering also individuals included in the first classification. In addition, COVID-19+ individuals were classified based on severity as mild (n = 11), moderate (n = 11), or severe (n = 5). The general characteristics of the study population are described in Table 1. As it is shown, several factors such as age, gender, obesity, hypertension, and previous heart disease are significant differential factors between groups. Thus, they were considered for further statistical analysis.

**Table 1:** General characteristics of the study population.

	COVID-19− (N = 36)	COVID-19+ (N = 27)	Mild disease (N = 11)	Moderate disease (N = 11)	Severe disease (N = 5)	Non-susceptible (N = 26)	Susceptible (N = 12)	*p*-value COVID-19+ vs. COVID-19−	*p*-value (susceptible vs. non-susceptible)
Age, median (P25–P75)	44 (24–84)	69 (41–92)	57 (50–75)	85 (75–89)	71 (59–92)	43 (28–60)	42 (30–58)	0.004	0.566
Sex, % women	26 (72)	14 (52)	5 (45)	6 (55)	3 (60)	20 (80)	8 (67)	0.097	0.351
Obese, N (%)	2 (6)	7 (26)	3 (27)	2 (18)	2 (40)	0 (0)	0 (0)	0.021	–
Hypertension, N (%)	2 (6)	15 (56)	6 (55)	5 (45)	3 (60)	0 (0)	0 (0)	< 0.001	–
Previous lung disease, N (%)	2 (6)	3 (11)	1 (9)	2 (18)	0 (0)	2 (8)	0 (0)	0.422	0.210
Previous heart disease, N (%)	1 (3)	6 (22)	2 (18)	2 (18)	2 (40)	0 (0)	0 (0)	0.013	–
Requiring supplemental oxygen at baseline, N (%)	–	11 (41)	0 (0)	6 (54)	5 (100)	–	–	–	–
Medications for COVID-19 during hospitalization*									
Azithromycin	–	13 (48)	5 (38)	5 (38)	3 (24)	–	–	–	–
Hydroxychloroquine	–	19 (70)	6 (32)	9 (47)	4 (21)	–	–	–	–
Lopinavir/ritonavir	–	13 (48)	1 (14)	9 (47)	3 (43)	–	–	–	–
Steroids	–	8 (30)	2 (25)	3 (38)	3 (38)	–	–	–	–
Tocilizumab	–	6 (100)	1 (17)	3 (50)	2 (33)	–	–	–	–
Clinical trial**	–	11 (41)	2 (18)	3 (27)	6 (55)	–	–	–	–

*The period of hospitalization was April-June 2020, during the first epidemic SARS-CoV-2 wave in Spain.

**Including aplidin (1 case, mild group), canakinumab (1 case, severe group), pirfenidone (1 case, severe group), remdesivir (1 case, severe group), sarilumab (1 case, severe group), hyperimmune serum (1 case, severe group).

### Untargeted lipidomics and metabolomics profiling of COVID-19 plasma samples

**Figure 1:**
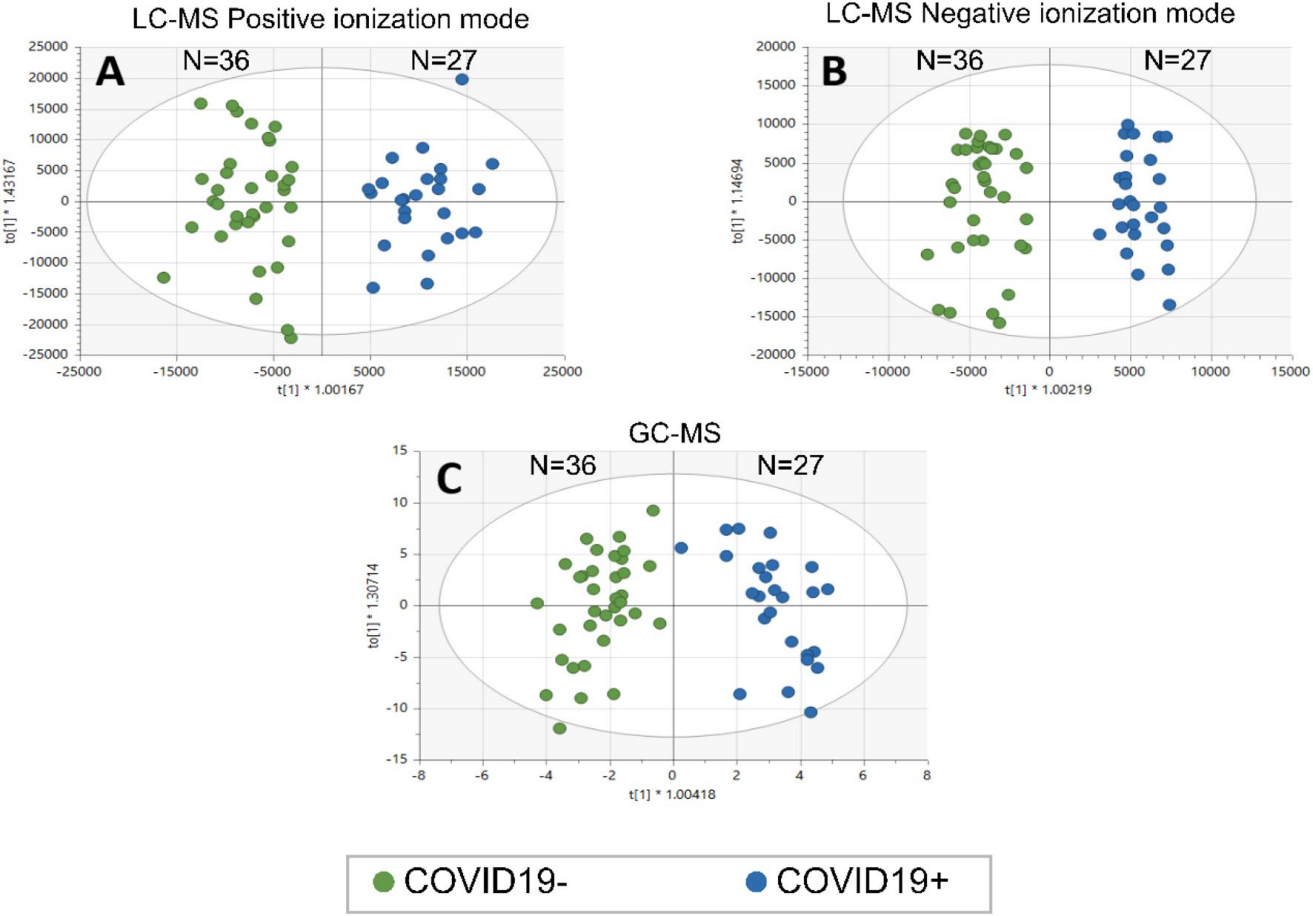
OPLS-DA scores plots obtained for COVID-19 positive and COVID-19 negative groups by different metabolomics platforms. (**A**) OPLS-DA score plot of LC–MS (ESI+). Statistics: R^2^ = 0.870, Q^2^ = 0.767, CV-ANOVA = 3.91e−15. (**B**) OPLS-DA score plot of LC–MS (ESI−). Statistics: R^2^ = 0.923, Q^2^ = 0.803, CV-ANOVA = 8.65e−16. (**C**) OPLS-DA score plot of GC–MS. Statistics: R^2^ = 0.880, Q^2^ = 0.715, CV-ANOVA = 1.66e−11.

Lipidomics analysis by LC–MS covering various lipid classes and metabolites identified by GC–MS led to the detection of several chromatographic signals that were compared by different statistical analyses for the groups COVID-19+ vs COVID-19− (Table S3-Sheet 1) and susceptible vs non-susceptible (Table S5-Sheet 2). For clarification and representation, OPLS-DA models were generated and validated to select the important variables that most influence group separation (Fig. 1). Furthermore, in order to correct any analytical variations and simplify visualization, QC-SVRC^28^, and PCA were performed (Fig. S1). As shown in the corresponding scores plots, a clear tendency and separation were observed in all cases. This analysis shows how COVID-19+ and COVID-19− groups are perfectly separated in both, LC–MS and GC–MS platforms, indicating that SARS-CoV2 infection impacts significantly the metabolomic and lipidomic profiles.

LC–MS allowed identifying differentially abundant in the COVID-19+ group compared with COVID-19− group. The lipid classes significantly obtained by univariate and multivariate statistical analysis that were identified from the total number in the positive (34%) and negative (29%) ionization modes, were classified as:: acylcarnitines (CAR), ceramides (Cer), hexosylceramides (HexCer), non-esterified fatty acids (FAs), fatty acid esters of hydroxy fatty acids (FAHFAs), phosphatidylcholines (PCs, PC-Os, LPCs, and LPC-Os), phosphatidylethanolamines (PEs, PE-Os, LPEs, and LPE-Os), phosphatidylinositols (PIs, and LPIs), phosphatidylserine (PSs), and sphingomyelins (SMs) (Fig. S2 and Table S3-Sheets 1–3). Additionally, depictions in heatmaps showed a clear separation of COVID-19+ and COVID-19− groups and revealed that Cer, PEs, and LPEs were upregulated in COVID-19+ patients. In contrast, all the other lipid classes were downregulated in COVID-19+ patients (Fig. S3).

**Figure 2:**
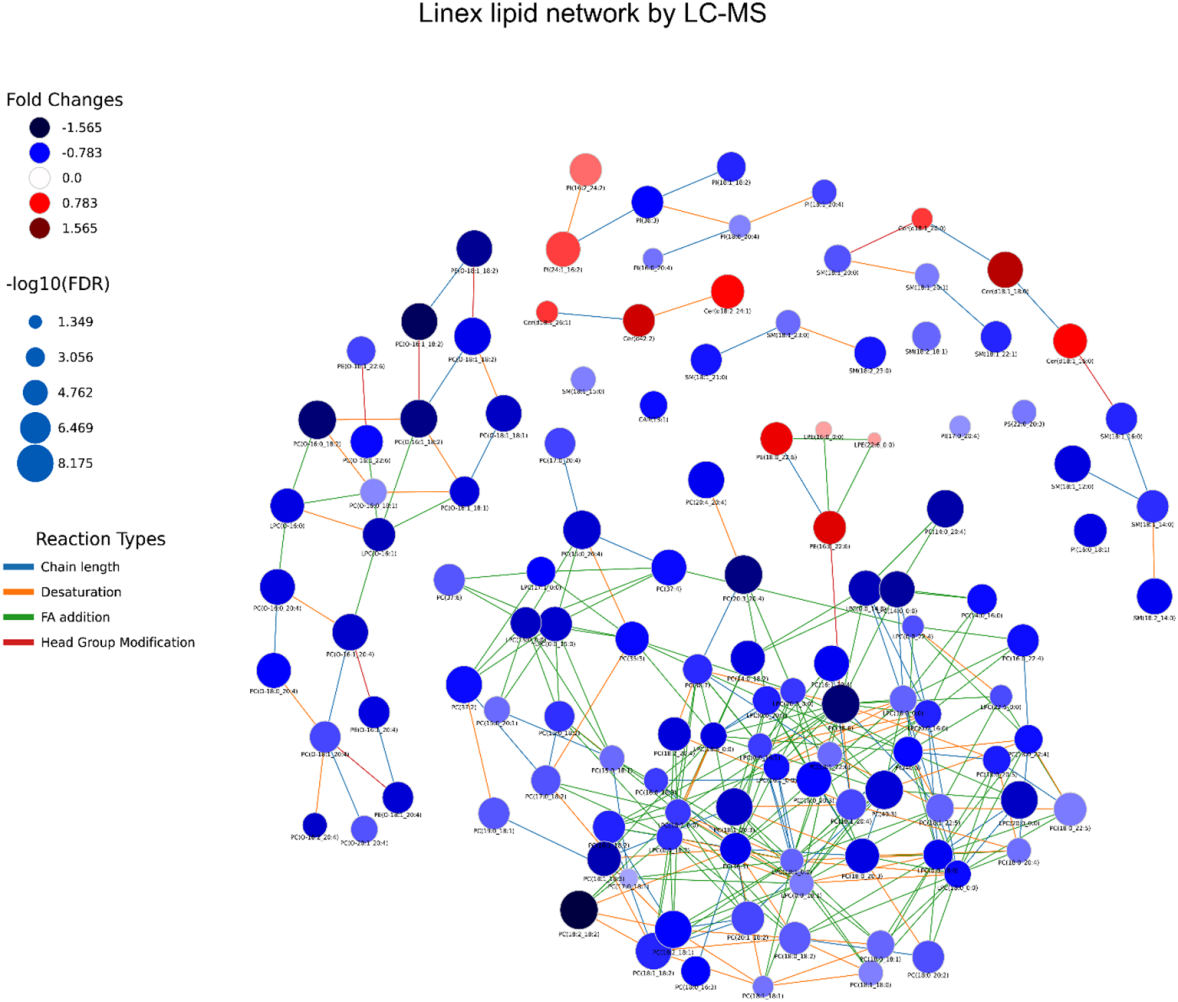
Lipid network of metabolites detected by LC–MS comparing COVID-19+ and COVID-19− groups and selecting the common hits identified after different statistical analyses. Lipid network connections were obtained by using Lipid Network Explorer software (LINEX, Version 1). Node colors represent the fold change obtained from LC–MS. Upregulated lipids in the COVID-19+ group are depicted in reddish tones in contrast to downregulated lipids that are depicted in bluish tones. Node size is -log10 (FDR), which is related to q-value, and edge color is the reaction type occurring between two lipids. 124 lipids were selected for being statistically significant by at least two statistical methods and they are further described in Table S3 Sheet LINEX_CommonSigMet.

Finally, we explored the lipid metabolic networks of all the lipids identified by LC–MS as significant by different statistical approaches and selected those common for two statistical methods (Fig. 2 and Table S3—LINEX_CommonSigMet sheet). The lipid network obtained is depicted in Fig. 2 and shows the differential detection of specific lipids in the COVID-19+ group, such as Cer, PI, and PE.

Similarly, by using GC–MS we identified several compounds from which 2-hydroxybutyric acid and 3-hydroxyisovaleric acid were found as significant metabolites by both, univariate and multivariate analysis (Mann–Whitney *U* and ANCOVA). A fold change of nearly 3 in both metabolites and effect sizes greater than 0.8 indicate these metabolites are highly upregulated in COVID-19+ individuals. Additionally, the comparison of COVID-19+ and COVID-19− groups gave 31 differential metabolites obtained by GC–MS, although, among them, only 7 remained statistically significant by at least two statistical methods (Fig. S3 and Table S4).

In summary, we found different groups of lipids and other metabolites when comparing patients divided by infection status (COVID-19+ vs COVID-19−). This pattern was shown by using the two methods, LC–MS and GC–MS, but was more evident for LC–MS, where all samples were perfectly clustered in different hierarchical branches.

### Metabolites related to COVID-19 severity

**Figure 3:**
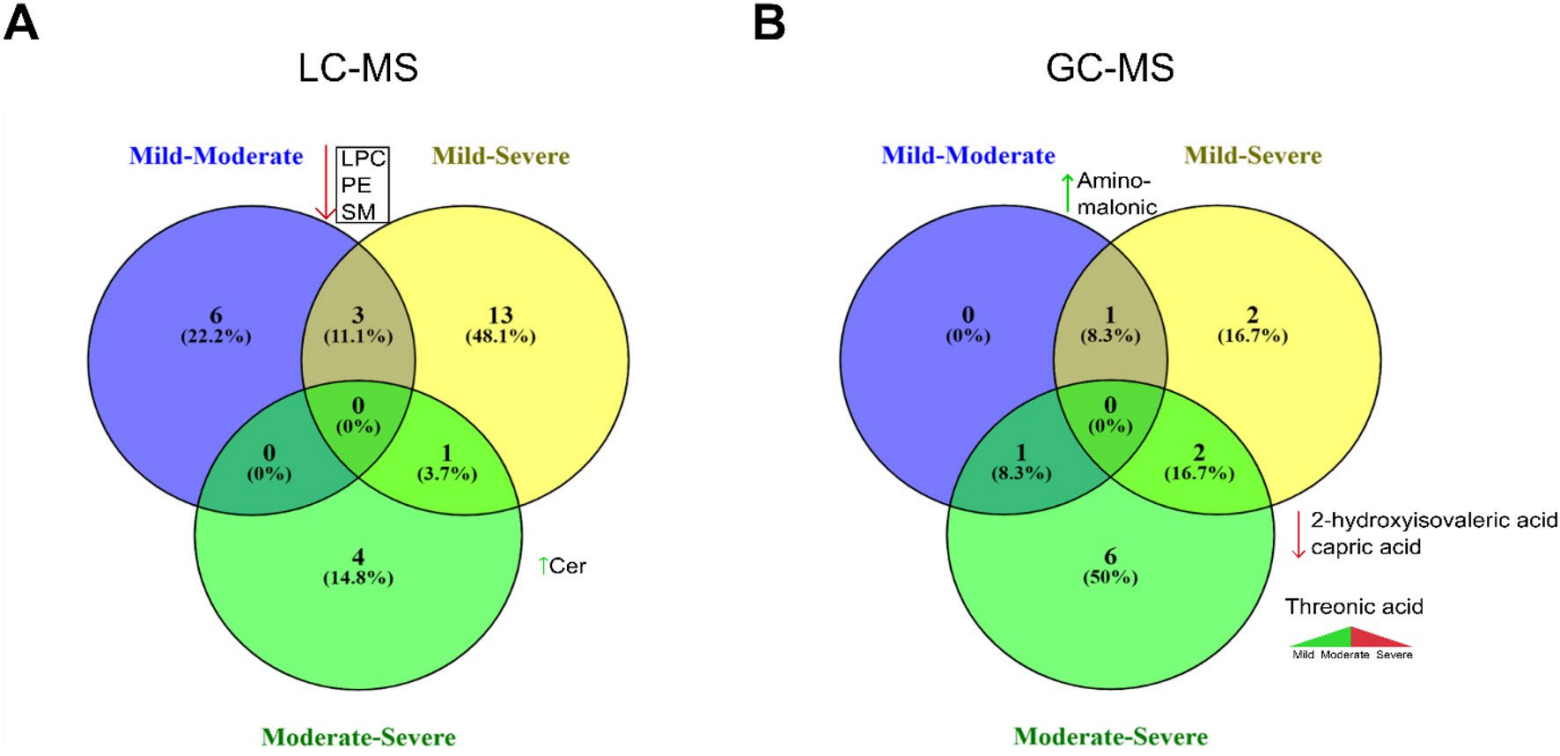
Venn diagram of the significant metabolites. Venn diagrams were obtained from the pairwise comparison (*q*-value ≤ 0.05) for (**A**) LC–MS and (**B**) GC–MS. The numbers indicate differentially expressed compounds in the more critical condition compared to the softer condition.

To explore the different symptoms and outcomes observed in COVID-19, we divided our samples for patients with confirmed SARS-CoV-2 into three groups as follows: 11 mild patients, 11 moderate, and 5 severe. Among the significant metabolites obtained in LC–MS, 9 identified lipids (*q*-value ≤ 0.05) differentiated between mild and moderate patients, 17 differentiated between mild and severe patients, and 5 differentiated between moderate and severe. Interestingly, 3 downregulated lipids [LPC (20:0/0:0), PE (O-18:2_20:4), and SM (18:1/20:0)] were common in the comparisons of mild vs moderate and mild vs severe, but not in moderate vs severe. Similarly, Cer (d42:2) is upregulated in severe patients compared to mild and moderate (Fig. 3A and Table S5).

In the case of GC–MS, 2 metabolites (*q*-value ≤ 0.05) differentiated between mild and moderate patients, 5 differentiated between mild and severe patients, and 9 differentiated between moderate and severe. Specifically, aminomalonic acid was found to be downregulated in severe patients compared to mild and moderate, whereas 2-hydroxyisovaleric acid and capric acid were upregulated in the severe group. Interestingly, analyzing the trend of threonic acid we observed that this metabolite is upregulated in the moderate group compared to the mild but then is downregulated in the severe group compared to the moderate group (Fig. 3B and Table S5).

### Differentially abundant (DA) lipid metabolites related to SARS-CoV-2 susceptibility

**Figure 4:**
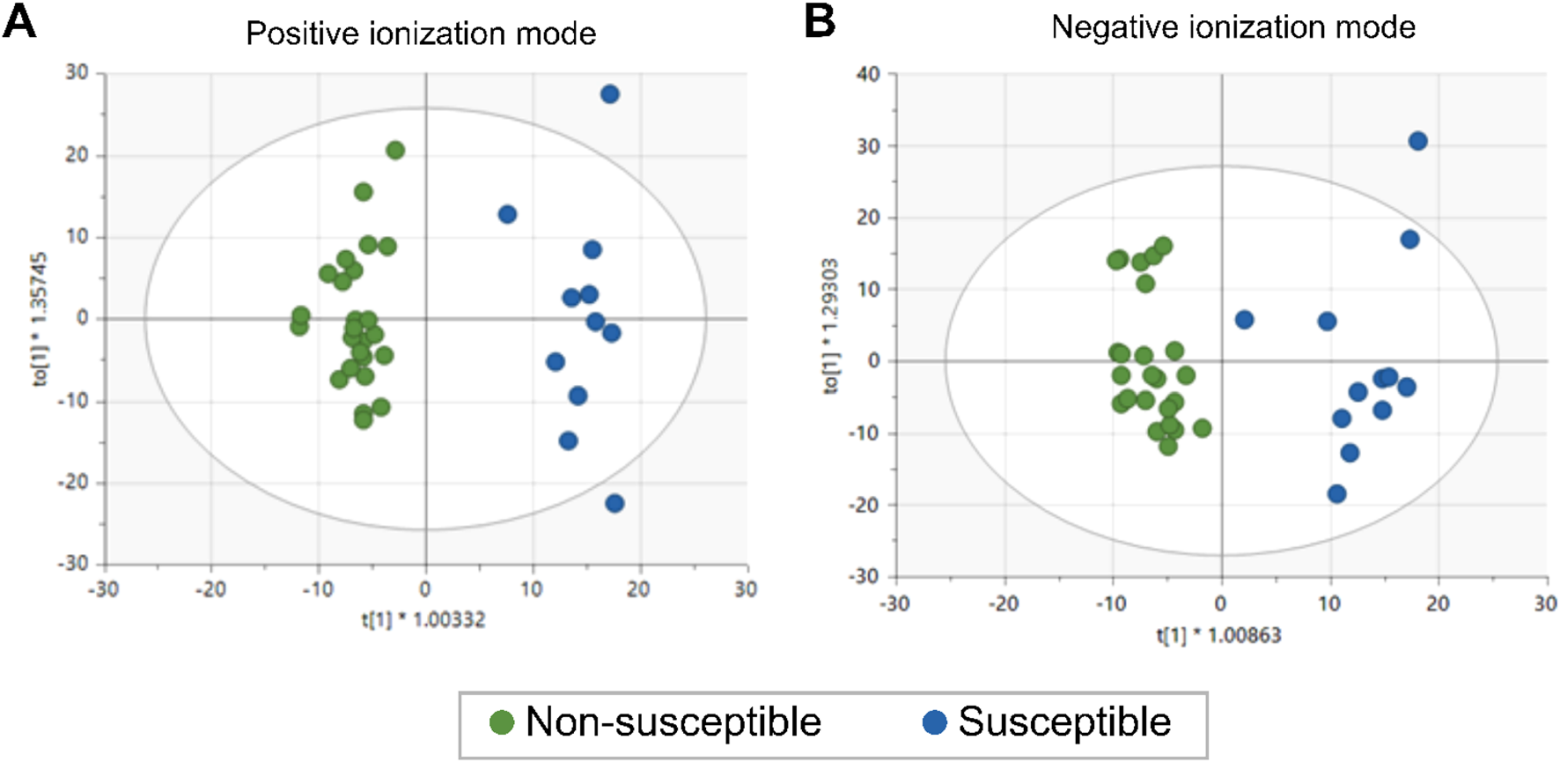
OPLS-DA scores plots representing lipid profiles from COVID-19 susceptible (n = 26) and non-susceptible (n = 12) individuals by LC–MS at positive and negative ionization modes. (**A**) OPLS-DA score plot of LC–MS (ESI+). Statistics: R^2^ = 0.945, Q^2^ = 0.699, CV-ANOVA = 6.79e−7. (**B**) OPLS-DA score plot of LC–MS (ESI−). Statistics: R^2^ = 0.903, Q^2^ = 0.687, CV-ANOVA = 3.17e−6.

A striking observation that remains unexplained is the different SARS-CoV-2 susceptibility across individuals. To shed some light on this question we analyzed samples from an exceptional group of healthcare workers heavily exposed to SARS-CoV-2 but not infected (named ‘non-susceptible’) compared to subjects who became infected during the follow-up (named ‘susceptible’). For this comparison, the pre-defined criteria for statistical validation (CV-ANOVA p-value ≤ 0.05) were met for models applied to LC–MS data (Fig. 4 and Fig. S4), but not for GC–MS data (plot C Fig. S4) although some common metabolites were found when comparing the two statistical methods (Fig. S5). Thus, we analyzed differentially abundant lipids by the LC–MS platform (Table S6) and performed multivariate and univariate statistical analyses, followed by QC-SVRC and PCA analysis using auto scaling (Fig. S4). Furthermore, we performed supervised methods and validated the PLS-DA and OPLS-DA models for LC–MS data. Since a clear separation is observed in the validated OPLS-DA analysis, this analysis suggests that SARS-CoV-2 susceptibility can be mainly differentiated by the lipidomic profiles (Fig. 4). This pattern is also shown when the DA lipids are depicted on heatmaps (Fig. S6).

**Figure 5:**
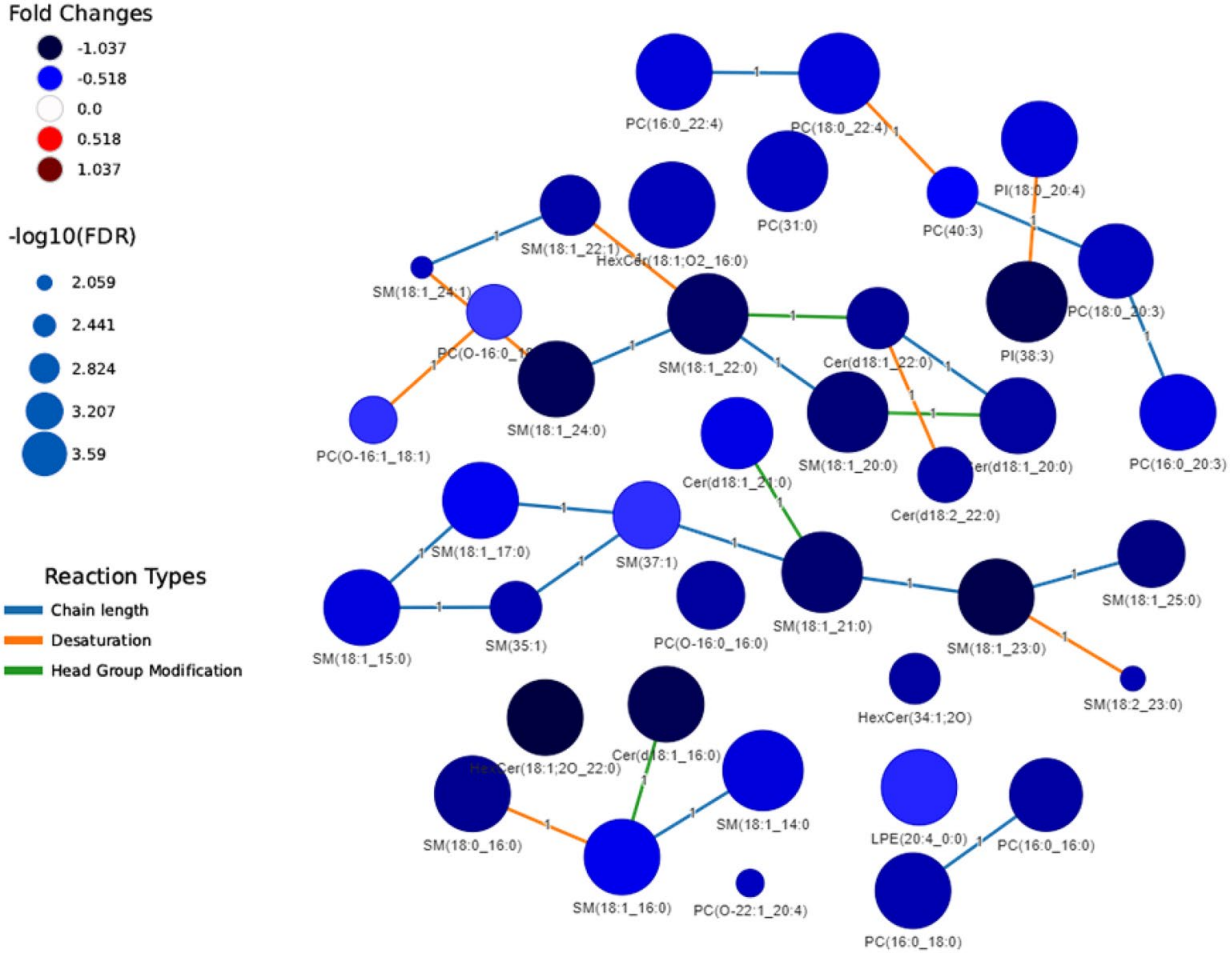
Lipid network connections generated by LINEX based on data from LC–MS and the comparison of susceptible vs non-susceptible groups common for at least two statistical analyses. Node colors represent the fold change of abundance obtained from LC–MS. Downregulated lipids in the susceptible group (i.e., upregulated in the non-susceptible group) are depicted in bluish tones. Node size is − log10 (FDR), which is related to the *q*-value of the lipid abundance, and edge color is the reaction type occurring between two lipids.

Finally, lipidomic network analyses revealed that all lipids were upregulated in non-susceptible individuals (Fig. 5, dark blue tonalities and Fig. S7). Among them, ceramides and sphingomyelins were highly upregulated, and PEs were not found significant in susceptible individuals. Furthermore, non-susceptible individuals grouped together and exhibited upregulation of specific lipidomic profiles compared to the non-susceptibles (Fig. S7 and Table S6).

## Discussion

In this study, we aimed at exploring the lipids and metabolites that could explain the differences in COVID-19 susceptibility. Our study includes a rare group of individuals, the non-susceptible participants, a small group of unvaccinated and heavily exposed healthcare workers who did not get infected by SARS-Cov-2. With the generalization of SARS-CoV-2 vaccines and the establishment of the pandemic with a wide circulation of SARS-CoV-2, this population is now exceptional and has become very hard to recruit, becoming increasingly rare. In our case, the early establishment of a sample collection of healthcare workers exposed to SARS-CoV-2 allowed us to include this group of individuals that can help understand the mechanisms driving SARS-CoV-2 susceptibility despite repeated virus exposures.

In the analysis of the effect of COVID-19 status on metabolomic profiles, we found clear differentiated lipidomics and metabolomics profiles for COVID-19+ patients compared to the non-infected group, as previously shown^4,33–36^. In the analysis by LC–MS, we observed that ceramides and phosphatidylethanolamines (PE) are upregulated in COVID-19+ patients compared to all the remaining lipid classes that are down-regulated. Ceramides and PE are involved in the maintenance of cell membrane structure and functions, influencing membrane-associated processes like cell signaling and immune responses^37–39^. Elevated ceramide levels has been found in plasma from COVID-19 patients^40–45^ and PE were enriched in analyzed samples from COVID-19 patients^46–48^. However, these studies are hardly comparable, since they have been carried out on different types of samples, different degree of disease progression, with variations in the techniques used, etc., so that causality cannot be established. Furthermore, in the analysis by GC–MS, 2-hydroxybutyric acid and 3-hydroxyisovaleric acid were found as significant metabolites, providing further support to previous studies that found α‑hydroxyl acids (AHAs) of amino acid origin increased with disease severity in COVID-19+ patients^49^. 2-hydroxybutyric acid is primarily produced in the process of L-threonine metabolism or glutathione synthesis. It may be increased by oxidative stress and is found in patients with energy metabolism deficiency^50^. Previous studies have also found 2-hydroxybutyric acid enriched in COVID-19 patients pointing an effect on the metabolism of these patients^51–53^. 3-Hydroxyisovaleric acid is an intermediate in the catabolic pathway of leucine. Other studies found increased levels of 3-hydroxyisovaleric acid in severe COVID-19 patients, suggesting COVID-19 may alter leucine metabolism or being a secondary consequence of an altered mitochondrial branched-chain amino acid (BCAA) metabolism related to hypoxia^54,55^, although more studies will be necessary to take this conclusion.

Several metabolomics studies have described specific lipids and metabolites patterns associated with COVID-19 severity. For example, a dysregulation of eicosanoids, variations in polyunsaturated fatty acids, key roles of tryptophan-nicotinamide pathway and cytosine metabolism or dysregulation of malic acid of the TCA cycle and carbamoyl phosphate of the urea cycle in patients with COVID-19 have been described^33,49,56,57^. In this sense, we also identified some specific compounds related to higher COVID-19 severity. In our data obtained by LC–MS, phospholipids and sphingomyelins are downregulated and ceramides upregulated in patients with more severe symptoms compared to those with less symptomatology. Furthermore, by GC–MS, aminomalonic acid is upregulated in milder conditions while 2-hydroxyisovaleric acid and capric acid were downregulated^58^. Although capric acid was identified in previous studies as having antiviral activity by disrupting the viral envelope^59^, there is no clear evidence on its role. One hypothesis will be that capric acid could be blocking the furin cleavage site specific in the SARS-CoV-2 spike protein, that allows the virus to infect the cells, thus impeding the viral entry and infectivity^60,61^. 2-hydroxyisovaleric which is also named 2-hydroxy-3-methyl butyric is an unsaturated acid that has been found overexpressed in patients who progressed from mild to severe symptoms during COVID-19^52,62^. Moreover, in this analysis, we see a compound, threonic acid, with dynamic behavior, being more abundant in the intermediate condition. The L-isomer of threonic acid is a metabolite of ascorbic acid (vitamin C)^63^ which has been inconclusively associated with infection, during which ROS are elevated, and specifically to COVID-19 severity, although this has not been fully confirmed^64,65^.

Finally, regarding susceptibility, a unique feature of our study population, we obtained differentially abundant lipids by LC–MS, highlighting the downregulation of lipids in general in the susceptible group, non-infected group, compared to the non-susceptible group. Specific profiles of sphingomyelins (such as SM 18:1/14:0, SM 18:1/16:1, and SM 18:1/18:0) and some ceramides (such as Cer d18:1/16:1, Cer d18:1/18:0, and Cer d18:1/22:0) have been associated to COVID-19 comorbidities such as obesity and cardiovascular-related conditions^66^. This coincides with the fact that lipid rafts are found to increase during viral infections^67^ since ACE2 can be mainly found within lipid rafts. Specifically, in our study, sphingomyelins and some specific ceramides were upregulated in the non-susceptible group. This might be due to a delocalization and deregulation of these lipids to the bloodstream instead of being destined for the formation of the lipid rafts in the cells, avoiding viral infection. This goes in agreement with previous studies where blocking of sphingomyelin/ceramide route of lipid rafts formation showed protection against SARS-CoV-2 infection^42^. Thus, dysregulations on lipid composition in blood could be considered as a marker for susceptibility to SARS-Cov-2 infection and the study of those could trigger to new antiviral strategies.

However, our study is subject to some limitations. The sample size was limited, due to the difficulties inherent in recruiting participants during the healthcare system overwhelming caused by the first SARS-CoV-2 pandemic waves, so the findings should be interpreted as exploratory. While our statistical modeling strategy included adjustment for the main confounding factors, as in any observational study, there is potential of unmeasured confounding. In addition, the study is limited to the first viral variant, so our findings cannot be extrapolated to current SARS-CoV-2 variants. Strengths of our study include the value of the non-susceptible population included, and the fact that we obtained the samples from patients with COVID-19+ early after symptom onset, implicating that the metabolites detected as markers of COVID-19 represent early metabolic consequences of the disease. Last, we used different untargeted metabolomics technologies that are complementary to each other, which allowed us to gain more resolution in finding metabolic markers of SARS-CoV-2 susceptibility and COVID-19 severity.

In conclusion, we found specific lipids profiles and metabolites explaining SARS-CoV-2 susceptibility and COVID-19 severity. Non-susceptible individuals showed a unique lipidomic pattern characterized by the upregulation of most lipids, especially ceramides and sphingomyelin, representing candidate predictors of susceptibility to SARS-CoV-2 infection. Since lipid composition can affect the cell membrane structure and virus infectivity, our study encourages further research on lipid profiles as relevant drivers of COVID-19 pathogenesis and encourages further research on the role of ceramides and sphingomyelin in the frontline against SARS-CoV-2 infection. Furthermore, since some of these compounds can be easily detected in clinical routine, the identification of relevant metabolites can be useful for the diagnosis and/or clinical management of COVID-19 patients. Consequently, lipidomics as well as lipids profiling is a powerful and relevant approach to improve compound coverage in COVID-19 disease.

### Supplementary Information

Supplementary Information.

## Data Availability

All data generated or analysed during this study are included in this published article (and its Supplementary Information files).

## References

[1] Wong JP, Damania B (2021). SARS-CoV-2 dependence on host pathways. Science.

[2] Gordon DE (2020). A SARS-CoV-2 protein interaction map reveals targets for drug repurposing. Nature.

[3] Chen Y (2020). Blood molecular markers associated with COVID-19 immunopathology and multi-organ damage. EMBO J..

[4] Gray N (2021). Diagnostic potential of the plasma lipidome in infectious disease: Application to acute SARS-CoV-2 infection. Metabolites.

[5] Chen MX, Wang S-Y, Kuo C-H, Tsai I-L (2019). Metabolome analysis for investigating host-gut microbiota interactions. J. Formos. Med. Assoc..

[6] Richards F (2022). Economic burden of COVID-19: A systematic review. Clin. Outcomes Res..

[7] Ma Y (2022). Long-term consequences of COVID-19 at 6 months and above: A systematic review and meta-analysis. Int. J. Environ. Res. Public. Health.

[8] Chen C (2022). Global prevalence of post-coronavirus disease 2019 (COVID-19) condition or long COVID: A meta-analysis and systematic review. J. Infect. Dis..

[9] Di Gennaro F (2022). Long covid: A systematic review and meta-analysis of 120,970 patients. SSRN Electron. J..

[10] Giamarellos-Bourboulis EJ (2020). Complex immune dysregulation in COVID-19 patients with severe respiratory failure. Cell Host Microbe.

[11] Vardhana SA, Wolchok JD (2020). The many faces of the anti-COVID immune response. J. Exp. Med..

[12] Merad M, Blish CA, Sallusto F, Iwasaki A (2022). The immunology and immunopathology of COVID-19. Science.

[13] Attaway AH, Scheraga RG, Bhimraj A, Biehl M, Hatipoğlu U (2021). Severe covid-19 pneumonia: pathogenesis and clinical management. BMJ.

[14] Shakaib B (2021). A comprehensive review on clinical and mechanistic pathophysiological aspects of COVID-19 Malady: How far have we come?. Virol. J..

[15] Bruzzone C, Conde R, Embade N, Mato JM, Millet O (2023). Metabolomics as a powerful tool for diagnostic, pronostic and drug intervention analysis in COVID-19. Front. Mol. Biosci..

[16] Danlos F-X (2021). Metabolomic analyses of COVID-19 patients unravel stage-dependent and prognostic biomarkers. Cell Death Dis..

[17] Wu D (2020). Plasma metabolomic and lipidomic alterations associated with COVID-19. Natl. Sci. Rev..

[18] Caterino M (2021). Dysregulation of lipid metabolism and pathological inflammation in patients with COVID-19. Sci. Rep..

[19] Valdés A (2022). Metabolomics study of COVID-19 patients in four different clinical stages. Sci. Rep..

[20] Dei Cas M (2021). Link between serum lipid signature and prognostic factors in COVID-19 patients. Sci. Rep..

[21] Hasan MR, Suleiman M, Pérez-López A (2021). Metabolomics in the diagnosis and prognosis of COVID-19. Front. Genet..

[22] Davis HE, McCorkell L, Vogel JM, Topol EJ (2023). Long COVID: Major findings, mechanisms and recommendations. Nat. Rev. Microbiol..

[23] Aguilar RB (2020). Current understanding of COVID-19 clinical course and investigational treatments. Front. Med..

[24] Albóniga OE (2022). Metabolic snapshot of plasma samples reveals new pathways implicated in SARS-CoV-2 pathogenesis. J. Proteome Res..

[25] Fanelli V (2013). Acute respiratory distress syndrome: New definition, current and future therapeutic options. J. Thorac. Dis..

[26] CDC, Centers for, disease control and prevention, & CDC. *Interim Operational Considerations for Public Health Management of Healthcare Workers Exposed to or with Suspected or Confirmed COVID-19: Non-U.S. Healthcare Settings*. https://www.cdc.gov/coronavirus/2019-ncov/hcp/non-us-settings/public-health-management-hcwexposed.

[27] Lejardi, D. & Technologies, A. *Improving Coverage of the Plasma Lipidome Using Iterative MS/MS Data Acquisition Combined with Lipid Annotator Software and 6546 LC/Q-TOF*. (2020).

[28] Kuligowski J, Sánchez-Illana Á, Sanjuán-Herráez D, Vento M, Quintás G (2015). Intra-batch effect correction in liquid chromatography-mass spectrometry using quality control samples and support vector regression (QC-SVRC). Analyst.

[29] Gil-de-la-Fuente A (2019). CEU mass mediator 3.0: A metabolite annotation tool. J. Proteome Res..

[30] Koelmel JP (2020). Lipid annotator: Towards accurate annotation in non-targeted liquid chromatography high-resolution tandem mass spectrometry (LC-HRMS/MS) lipidomics using a rapid and user-friendly software. Metabolites.

[31] Tsugawa H (2020). A lipidome atlas in MS-DIAL 4. Nat. Biotechnol..

[32] Köhler N, Rose TD, Falk L, Pauling JK (2021). Investigating global lipidome alterations with the lipid network explorer. Metabolites.

[33] Sindelar M (2021). Longitudinal metabolomics of human plasma reveals prognostic markers of COVID-19 disease severity. Cell Rep. Med..

[34] Schmelter F (2021). Metabolic and lipidomic markers differentiate COVID-19 from non-hospitalized and other intensive care patients. Front. Mol. Biosci..

[35] Žarković N (2022). Lipidomics revealed plasma phospholipid profile differences between deceased and recovered COVID-19 patients. Biomolecules.

[36] Bizkarguenaga M (2022). Uneven metabolic and lipidomic profiles in recovered COVID-19 patients as investigated by plasma NMR metabolomics. NMR Biomed..

[37] Albeituni S, Stiban J, Honn KV, Zeldin DC (2019). Roles of ceramides and other sphingolipids in immune cell function and inflammation. The Role of Bioactive Lipids in Cancer, Inflammation and Related Diseases.

[38] Gómez-Muñoz A (2005). Ceramide-1-phosphate promotes cell survival through activation of the phosphatidylinositol 3-kinase/protein kinase B pathway. FEBS Lett..

[39] Hou TY (2016). n-3 polyunsaturated fatty acids suppress CD4+ T cell proliferation by altering phosphatidylinositol-(4,5)-bisphosphate [PI(4,5)P2] organization. Biochim. Biophys. Acta BBA Biomembr..

[40] Khodadoust MM (2021). Inferring a causal relationship between ceramide levels and COVID-19 respiratory distress. Sci. Rep..

[41] Alexander MP (2021). Acute kidney injury in severe COVID-19 has similarities to sepsis-associated kidney injury. Mayo Clin. Proc..

[42] Kornhuber J, Hoertel N, Gulbins E (2022). The acid sphingomyelinase/ceramide system in COVID-19. Mol. Psychiatry.

[43] Petrache I (2023). Marked elevations in lung and plasma ceramide in COVID-19 linked to microvascular injury. JCI Insight.

[44] Törnquist K, Asghar MY, Srinivasan V, Korhonen L, Lindholm D (2021). Sphingolipids as modulators of SARS-CoV-2 infection. Front. Cell Dev. Biol..

[45] Song J-W (2020). Omics-driven systems interrogation of metabolic dysregulation in COVID-19 pathogenesis. Cell Metab..

[46] Byeon SK (2022). Development of a multiomics model for identification of predictive biomarkers for COVID-19 severity: A retrospective cohort study. Lancet Digit. Health.

[47] Kurano M (2022). Dynamic modulations of sphingolipids and glycerophospholipids in COVID-19. Clin. Transl. Med..

[48] Ren Z (2021). Alterations in the human oral and gut microbiomes and lipidomics in COVID-19. Gut.

[49] Páez-Franco JC (2021). Metabolomics analysis reveals a modified amino acid metabolism that correlates with altered oxygen homeostasis in COVID-19 patients. Sci. Rep..

[50] Cobb J (2016). α-Hydroxybutyric acid is a selective metabolite biomarker of impaired glucose tolerance. Diabetes Care.

[51] Casari I, Manfredi M, Metharom P, Falasca M (2021). Dissecting lipid metabolism alterations in SARS-CoV-2. Prog. Lipid Res..

[52] Shi D (2021). The serum metabolome of COVID-19 patients is distinctive and predictive. Metabolism.

[53] Kumar R, Kumar V, Arya R, Anand U, Priyadarshi RN (2022). Association of COVID-19 with hepatic metabolic dysfunction. World J. Virol..

[54] Torres-Ruiz J (2021). Redefining COVID-19 severity and prognosis: The role of clinical and immunobiotypes. Front. Immunol..

[55] Barberis E (2020). Large-scale plasma analysis revealed new mechanisms and molecules associated with the host response to SARS-CoV-2. Int. J. Mol. Sci..

[56] Barberis E (2020). Large-scale plasma analysis revealed new mechanisms and molecules associated with the host response to sars-cov-2. Int. J. Mol. Sci..

[57] Torretta E (2021). Severity of COVID-19 patients predicted by serum sphingolipids signature. Int. J. Mol. Sci..

[58] HMDB. *Human Metabolome Database: Showing metabocard for Aminomalonic acid (HMDB0001147)*. https://hmdb.ca/metabolites/HMDB0001147.

[59] Hierholzer JC, Kabara JJ (1982). In vitro effects of monolaurin compounds on enveloped RNA and DNA viruses. J. Food Saf..

[60] Johnson BA (2021). Loss of furin cleavage site attenuates SARS-CoV-2 pathogenesis. Nature.

[61] Essalmani R (2022). Distinctive roles of furin and TMPRSS2 in SARS-CoV-2 infectivity. J. Virol..

[62] Gomez-Gomez A (2022). Untargeted detection of the carbonyl metabolome by chemical derivatization and liquid chromatography-tandem mass spectrometry in precursor ion scan mode: Elucidation of COVID-19 severity biomarkers. Anal. Chim. Acta.

[63] HMDB. *Human Metabolome Database: Showing Metabocard for Threonic acid (HMDB0000943)*. https://hmdb.ca/metabolites/HMDB0000943.

[64] Milani GP, Macchi M, Guz-Mark A (2021). Vitamin C in the treatment of COVID-19. Nutrients.

[65] Toscano GAS, de Araújo II, de Souza TA, Barbosa Mirabal IR, de Vasconcelos Torres G (2021). Vitamin C and D supplementation and the severity of COVID-19: A protocol for systematic review and meta-analysis. Medicine.

[66] Kočar E, Režen T, Rozman D (2021). Cholesterol, lipoproteins, and COVID-19: Basic concepts and clinical applications. Biochim. Biophys. Acta BBA Mol. Cell Biol. Lipids.

[67] Suzuki T, Suzuki Y (2006). Virus infection and lipid rafts. Biol. Pharm. Bull..

