# Author Correction: Differential abundance of lipids and metabolites related to SARS-CoV-2 infection and susceptibility

**DOI:** 10.1038/s41598-023-47669-6

**Published:** 2023-12-18

**Authors:** Oihane E. Albóniga, Elena Moreno, Javier Martínez-Sanz, Pilar Vizcarra, Raquel Ron, Jorge Díaz-Álvarez, Marta Rosas Cancio-Suarez, Matilde Sánchez-Conde, Juan Carlos Galán, Santiago Angulo, Santiago Moreno, Coral Barbas, Sergio Serrano-Villar

**Affiliations:** 1 Centro de Metabolómica y Bioanálisis (CEMBIO), Facultad de Farmacia, Universidad San Pablo-CEU, CEU Universities, Urbanización Montepríncipe, Boadilla del Monte, 28660 Madrid, Spain; 2 Department of Infectious Diseases, Hospital Universitario Ramón y Cajal, IRYCIS, 28034 Madrid, Spain; 3 CIBERINFEC, Instituto de Salud Carlos III, Madrid, Spain; 4 Department of Microbiology, Hospital Universitario Ramón y Cajal, IRYCIS, 28034 Madrid, Spain; 5 CIBERESP, Instituto de Salud Carlos III, Madrid, Spain; 6 Department of Infectious Diseases, Hospital Universitario Ramon y Cajal, Facultad de Medicina, Universidad de Alcalá (IRYCIS), Carretera de Colmenar Viejo, Km 9.100, 28034 Madrid, Spain

Correction to: *Scientific Reports* 10.1038/s41598-023-40999-5, published online 13 September 2023

The original version of this Article contained an error in the spelling of the author Marta Rosas Cancio-Suarez which was incorrectly given as Marta Rosas.

The original Article has been corrected.

