# Supplementary material for: Differential abundance of lipids and metabolites related to SARS-CoV-2 infection and susceptibility
Source: Sci Rep. 2023 Sep 13;13:15124. doi: 10.1038/s41598-023-40999-5 (PMC10500013; doi:10.1038/s41598-023-40999-5)
Supplement: Supplementary file 1 — Supplementary Information. [file 41598_2023_40999_MOESM1_ESM.zip › ALL_Sup_Info_SR_May23/Sup_Info_Differential_abundance_of_lipids_SR_reviewed July 23.docx]

**Supplementary Information For:**

**Differential abundance of lipids and metabolites related to SARS-CoV-2 infection**

**Oihane E. Albóniga^1†^, Elena Moreno^2,3†^, Javier Martínez-Sanz^2,3^, Pilar Vizcarra^2,3^, Raquel Ron^2,3^, Jorge Diaz^2,3^, Marta Rosas Cancio-Suarez^2,3^, Matilde Sánchez-Conde^2,3^, Juan Carlos Galán^3,4^,** **Santiago Angulo^1^, Santiago Moreno^2,3^, Coral Barbas^1,§^, Sergio Serrano-Villar^2,3,§^**

^1^Centro de Metabolómica y Bioanálisis (CEMBIO), Facultad de Farmacia, Universidad San Pablo-CEU, CEU Universities, Urbanización Montepríncipe, Boadilla del Monte, 28660 Madrid, Spain.

^2^Department of Infectious Diseases, Hospital Universitario Ramón y Cajal, IRYCIS, 28034, Madrid, Spain.

^3^CIBERINFEC, Instituto de Salud Carlos III, Madrid, Spain

^4^Department of Microbiology, Hospital Universitario Ramón y Cajal, IRYCIS, 28034, Madrid, Spain.

**† These authors have contributed equally to this work and share the first authorship**

**^§^ These authors share senior authorship**

**Correspondence:**Sergio Serrano-Villar, MD, PhD. Department of Infectious Diseases, Hospital Universitario Ramon y Cajal, Facultad de Medicina, Universidad de Alcalá (IRYCIS). Carretera de Colmenar Viejo, Km 9.100, 28034 Madrid, Spain. E-mail: [sergio.serrano@salud.madrid.org](mailto:sergio.serrano@salud.madrid.org)

Coral Barbas. Centro de Metabolómica y Bioanálisis (CEMBIO), Facultad de Farmacia, Universidad San Pablo-CEU, CEU Universities, Urbanización Montepríncipe, Boadilla del Monte, 28660 Madrid, Spain. Email: cbarbas@ceu.es

**Table of contents**

**Extra information about methods**

**Supplementary figures:**

**Figure S1. PCA-X and PLS-DA scores plots obtained for COVID-19 positive and COVID-19 negative groups by different metabolomics platforms.**

**Figure S2. Common significant metabolites obtained by LC-MS and comparing COVID-19+ and COVID-19- groups.**

**Figure S3. Significant metabolites obtained by GC-MS.**

**Figure S4**. **PCA-X and PLS-DA scores plots obtained for susceptible and non-susceptible groups by different metabolomics platforms.**

**Figure S5.** **Significant metabolites obtained by GC-MS comparing susceptible and non-susceptible groups.**

**Figure S6. Heatmaps obtained using the significant metabolites obtained by LC-MS and comparing susceptible and non-susceptible groups.**

**Figure S7. Lipid network connections generated by LINEX based on data from LC-MS and the comparison of susceptible vs non-susceptible groups.**

**Supplementary tables:**

**Table S1. Total data matrix from all metabolomics techniques used.**

**Table S2**. **Common metabolites found to be statistically significant from multivariate statistical analysis (MVDA).**

**Table S3***.* **Differential metabolites found by LC-MS and significant for COVID-19 disease (COVID-19+ vs COVID-19-).**

**Table S4.** **Metabolites found by GC-MS and significant for COVID-19 disease (COVID-19+ vs COVID-19**

**Table S5. Differential metabolites found by LC-MS and significant when two-by-two comparisons are performed using different disease progression states (mild, moderate, and severe).**

**Table S6. Differential metabolites found by LC-MS and significant for susceptible vs non-susceptible comparison.**

**Extra information about methods:**

**Chemical Reagents**

The aqueous solutions were prepared using reverse-osmosed ultrapure water obtained ‘in-house’ from a Milli-Qplus185 system (Millipore, Billerica, MA, SA). LC-MS grade methanol (MetOH), acetronitrile (ACN), and isopropanol (IPA) were purchased from Fisher Scientific (Pennsylvania, United States). Ammonium fluoride (NH_4_F) (ACS reagent, ≥ 98%) was obtained from Sigma-Aldrich (Steinheim, Germany), and analytical grade ammonia solution (28%, GPR RECTAPUR®) as well as acetic acid glacial (AnalaR® NORMAPUR®) were obtained from VWR Chemicals (Pennsylvania, United States). The internal standards (IS) used in GC-MS (palmitic acid – d31 and tricosane) and reagents for derivatization (O-methoxyamine hydrochloride and BSTFA:TMCS, 99:1 (Sylon BFT)) were purchased from Sigma-Aldrich. Two standard mixes for GC-MS, one containing grain fatty acid methyl esters (C8:0-C22:1, n9) and another standard mix with a mixture of n-alkanes (C8-C40) were obtained from Fluka Analytical (Sigma-Aldrich Chemie GmbH, Steinheim, Germany). Finally, n-heptane, 99% and sylilation-grade pyridine were obtained from Carlo Erba Reagents-SA (DASITGROUP, Spain) and AnalaR® NORMAPUR® (VWR Chemicals, Pensylvannia, United States), respectively.

**Plasma fingerprinting by LC-MS and GC-MS**

Plasma samples were inactivated and mixed with Ethanol:Methanol (see main text) were vortex-mixed for 1 min, incubated on ice for 5 min, and centrifuged at 12,500 rpm for 20 min at 4 ˚C in order to precipitate and remove proteins. The clean upper layer or supernatant, which contained the metabolites of interest, was transferred to Eppendorf tubes and stored at -80 ˚C until analysis. 200 µL of frozen supernatant was thawed on ice for sample preparation. The processes were adapted from previously reported protocols [1,2].

***Sample preparation and analysis for LC-MS***

For LC-MS, 200 µL of defrosted plasma supernatant were centrifuged for 10 min at 13,200 rpm at 4 ℃ and transferred to an LC-MS vial for analysis 200 µL of each plasma supernatant was thawed on ice to room temperature and centrifuged for 10 min at 13,200 rpm at 4 °C, transferred to an LC-MS vial and directly injected into the system. The lipidomics data were acquired using an Agilent 1290 Infinity II UHPLC system coupled to an Agilent 6545 quadrupole time-of-flight (QTOF) mass spectrometer and equipped with an electrospray ionization source (ESI) operating in positive and negative modes.

The analysis was performed using a previously reported method [3] by injecting 1 µL of sample on an Agilent InfinityLab Poroshell 120 EC-C18 (3.0 x 100mm, 2.7 µm) equipped with a guard column (Agilent InfinityLab Poroshell 120 EC-18, 3.0 x 5 mm, 2.7 µm), both from Agilent Technologies, at 50 °C and 0.6 mL/min. The mobile phases for both, positive and negative ionization modes, consisted of (A) 10 mM ammonium acetate, 0.2 mM ammonium fluoride in 9:1 water/MeOH, and (B) 10 mM ammonium acetate, 0.2 mM ammonium fluoride in 2:3:5 acetonitrile/MeOH/isopropanol. The chromatographic gradient started at 70 % of B at 0 – 1 min, 86 % B at 3.5 – 10 min, and 100% B at 11 – 17 min. The starting conditions were recovered by minute 17, followed by a 2 min re-equilibration time, reaching a total running time of 19 min. The multiwash strategy consisted of a mixture of MeOH:IPA (50:50, v/v) with the wash time set at 15 s, and aqueous phase:organic phase (30:70, v/v) mixture to assist in the starting conditions.

The Agilent 6545 QTOF mass spectrometer parameters, equipped with a dual AJS ESI ion source, were as follows: 150 V fragmentor, 65 V skimmer, 3500 V capillary voltage, 750 V octopole radio frequency voltage, 10 L/min nebulizer gas flow, 200 °C gas temperature, 50 psi nebulizer gas pressure, 12 L/min sheath gas flow, and 300 °C sheath gas temperature. Data in positive and negative ionization modes were acquired in separate runs, operated in full scan mode from 50 to 1800 m/z with a scan rate of 3 spectra/s. A solution containing two reference mass compounds was used throughout the whole analysis: purine (C_5_H_4_N_4_) at *m/z* 121.0509 for the positive and *m/z* 119.0363 for the negative ion modes; and HP-0921 (C_18_H_18_O_6_N_3_P_3_F_24_) at *m/z* 922.0098 for the positive and *m/z* 1033.9881 for the negative ionization modes. The solution with reference masses was infused continuously into the system through an Agilent 1260 IsoPump at 1 mL/min (split ratio 1:100) to provide a constant mass correction. Ten iterative MS/MS runs were performed also for positive and negative ionization modes at the end of the analytical sequence in a QC sample. They were operated with an MS and MS/MS scan rates of 3 spectra/s, 100-1700 m/z mass range, a narrow (~ 1.3 amu) MS/MS isolation width, 3 precursors per cycle, and 500 counts and 0.001 % of MS/MS threshold. Five iterative MS/MS runs were set with a collision energy of 20 eV, and the subsequent five runs were performed at 40 eV. Reference masses and contaminants detected in blank samples were excluded from the analysis to avoid their inclusion in the iterative MS/MS.

**Sample preparation and analysis for GC-MS**

200 µL of each plasma supernatant was thawed on ice to room temperature and 30 µL of palmitic acid – d31 in MeOH (80 mg/mL) was added. Samples were vortex mixed for 5 min and 200 µL were transferred to a GC-MS vial. Then, samples were evaporated to dryness using a SpeedVac Concentrator System (Thermo, Fisher Scientific, Waltham, MA) and maintained in the garage at 8 °C in the Gerstel Multiple Purpose Sample (MPS) Preparation Station (GERSTEL, Inc., Maryland, USA). An automated two-step derivatization process was performed prior to sample injection using an adjusted protocol from a previously reported method [4]. First, each precipitate was redissolved in 20 µL of *O*-methoxyamine solution (15 mg/mL in pyridine) for the methoximation process, mixed for 10 min at 1,000 rpm, and incubated for 90 min at 60 °C at 750 rpm. Second, and after waiting 5 min in the garage, 40 µL of BSTFA with 1% TMCS were added for the silylation process. Then, samples were mixed for 10 min at 1,000 rpm and incubated for 60 min at 60 °C at 750 rpm. After waiting 30 min at 8 °C, 80 µL of heptane containing 20 mg/mL of tricosane (IS) was added and mixed for 5 min at 1,000 rpm. Finally, samples were maintained at 8 °C for 30 min prior to injection. The total runtime for each sample preparation was 42 min.

Derivatized samples were analyzed in a GC-MS system (8890) coupled to a single quadrupole mass spectrometer (5977B), both from Agilent Technologies. 2 µL of derivatized plasma samples were automatically injected in split mode (split ratio 1:10) by an Agilent autosampler (7693) into an Agilent ultra-inert deactivated glass wool split liner. Metabolite separation was achieved using a pre-column (10 m J&W integrated with Agilent 122-5532G) combined with a GC DB5-MS column (length, 40 m; inner diameter, 0.25 mm; and 0.25 µm film of 95% dimethyl/5% diphenylpolysiloxane). The flow rate of helium carrier gas was constant at 0.5658 mL/min through the column. The retention time (RT) was locked according to the peak of the internal standard C18 (methyl stearate) at 19.66 minutes. The oven temperature gradient was initially set at 60 °C for 1 min. Then, it was raised by 10 °C/min until it reached 325 °C, and then it was held at this temperature for 10 min before cooling down. The total run time was 37 min followed by 5 min of post-run. The injector and transfer line temperatures were set at 250 °C and 280 °C, respectively. The operating parameters of the electron ionization (EI) source were set as follows: filament source temperature at 230 °C and electron ionization energy at 70 eV. Mass spectra were collected in a mass range from 50 to 600 at a scan rate of 2 spectra/s. Data were acquired using the Agilent MassHunter Workstation GC-MS Data Acquisition (version 10.0). To determine the retention rate, a mixture of n-alkanes (C8-C28) dissolved in n-hexane was analyzed before the samples. Quality control samples (QC) were prepared by pooling and mixing equal volumes of each plasma sample supernatant and were treated as previously described for LC-MS and GC-MS. Blank solutions were also prepared with MeOH:EtOH (1:1, v/v) and treated following the previous protocols.

**Data Processing**

***LC-MS data treatment***

Acquired raw data, in positive and negative ionization modes, were checked using MassHunter Qualitative software (version 10.0) to determine the data quality, the system mass accuracy, and the reproducibility of QC injections. Then, raw data were processed with the Agilent MassHunter Profinder software (version B.10.0.2) applying a two steps process, first the Batch Molecular Feature Extraction (MFE) and second the Batch Recursive Feature Extraction (RFE) algorithms. The MFE algorithm was used to clean the data background and unrelated ions or unwanted information. MFE finally creates a list of possible components that represent the full range of time-of-flight (TOF) mass spectral data features, which are the sum of coeluting ions that are related by charge-state envelope, isotopologue pattern, and/or the presence of different adducts and dimers. Afterward, the MFE detects coeluting adducts of the same feature, selecting the following adduct [3]: [M+H]^+^, [M+C_2_H_6_N_2_+H]^+^, [M+Na]^+^, [M+K]^+^ and [M+NH_4_]^+^ in LC-MS positive ionization; [M-H]^-^, [M+CH_3_COOH-H]^-^, [M+CH_3_COONa-H]^-^, and [M+Cl]^-^ in LC-MS negative ion mode. The neutral loss (NL) of water was also considered for both ionizations. Sn-1 and sn-2 lyso-phospholipids were differentiated based on the relative abundances of the fragments 184 and 104 that are common fragments of lyso PCs, based on their RT (slightly different between sn-1 and sn-2), and based on the fact that sn-2 elute earlier than sn-1. Finally, all the molecular features across the study samples were aligned by using the mass and retention time (RT) to build a single spectrum for each compound group. The next step involves the RFE algorithm, using the median values derived from the MFE process to perform a targeted extraction to improve the reliability of finding and reporting features from complex datasets used for differential analysis [5]. The reference masses (*m/z* 121.0509 and *m/z* 922.0098, in positive ionization, and *m/z* 112.0508 and *m/z* 1033.9881, in negative ionization) infused and monitored during the analysis as well as those features found in blanks, were excluded from the final list. Finally, the data matrix was imported in Microsoft Excel and filtered before statistical analysis as follows: (1) metabolites not presented in 70% of samples in at least one sample group were excluded, (2) missing values obtained from the RFE algorithm were filled by *k*-nearest neighbor (KNN) algorithm, (3) metabolites presented in blanks, and (4) metabolites with a percentage of coefficient of variation (% CV) in the QCs greater than 30% were removed.

***GC-MS data treatment***

Similarly to LC-MS, GC-MS data were checked using MassHunter Qualitative software (version 10.0) to assess the analytical performance, the data quality, the QCs injection reproducibility, and the IS signal by analyzing the total ion chromatogram (TIC) obtained for each sample, blanks, and QCs. Then, raw data files were imported into MassHanter Quantitative Unknowns Analysis software (version 10.0) to perform the deconvolution and identification of the metabolites by searching into two target libraries: Fiehn library (version 2008) and the ‘in-house’ plasma library built in CEMBIO based on Fiehn, as well as in NIST (National Institute of Standards and Technology, library 2.2 version 2014) libraries. Afterward, data obtained were aligned in the Agilent Mass Profiler Professional (version 15.1) and exported into the Agilent MassHunter Quantitative Analysis (version 10.0) to assign target ions and obtain the compound abundances. The correct integration of the peaks was inspected and the data matrix was generated. Finally, the generated matrix with the final abundances of each metabolite was treated following the same workflow mentioned in LC-MS data treatment and, in this case, the data matrix was also normalized according to the IS abundance (palmitic acid - d31) prior to any statistical analysis.

**Statistical Analysis**

Multivariate (MVDA) and univariate (UVDA) statistical analyses were carried out to find those metabolites that significantly differentiate groups. Different comparisons were performed to evaluate COVID-19 disease (COVID-19- and COVID-19+), susceptibility (non-susceptible and susceptible), and disease severity (mild, moderate, and severe). Furthermore, and considering the small sample size, severity was studied with COVID-19+ samples distributed as mild, moderate, and severe individuals as a tentative and initial approach for future studies. The filtered matrix obtained in the previous step was processed by SIMCA-P version 16.0.1 (Umetrics, Umea, Sweden), MATLAB software (R2018b, The MathWorks, Maticks, MA, USA), MetaboAnalyst 5.0 and/or SPSS version 27 (IBM SPSS Statistics). The intensity drop was corrected with the intra-batch effect correction using the QCs and support vector regression (QC-SVRC) [6].

For MVDA, SIMCA-P software was used for both unsupervised (principal component analysis, PCA) and supervised analysis (partial least-squares discriminant analysis, PLS-DA, or orthogonal partial least-squares discriminant analysis, OPLS-DA). PCA was performed to reduce dimensionality and to study the data quality, assess the reliability of the analytical procedure, visualize the natural grouping of the samples and determine the presence of outliers. Samples out of Hotelling’s T2 (95% confidence level) as well as QC samples were removed for further supervised analysis. Model quality was first analyzed by the explained variance (R^2^) and the predicted variance (Q^2^). R^2^ and Q^2^ had to be greater than 0.6 and 0.4, respectively, or the difference between them was less than 0.3 [7]. Afterward, PLS-DA and OPLS-DA were performed to maximize differences between the groups. After a suitable model validation by cross-validated analysis of variance (CV-ANOVA) [7], the OPLS-DA model was used for variable selection. The criteria metabolites must fulfill was a variable influence on projection (VIP) score greater than 1.0 and an absolute value of p(corr) greater than 0.6.

For UVDA, the Mann-Whitney U test (*p*-value ≤0.05), performed in Matlab (R2018b, The MathWorks, Maticks, MA, USA), for COVID-19 disease and susceptibility was used, as well as analysis of covariance (ANCOVA, the *p*-value for interesting label ≤0.05), carried out in SPSS (IBM SPSS Statistics 27), when the confounding factors were found to be significant among groups (see Table 1 in the Manuscript). In both cases, the false discovery rate at a level of α = 0.05 was controlled by the Benjamini-Hochberg correction test (*q*-value ≤ 0.05). Both statistical methods and the significant metabolites obtained were considered for biological interpretation as these reveal a broader range for biological interpretation and consequently pathophysiological alterations. Finally, the means and standard deviations (or their adjusted values) were used to calculate the fold-change (FC) and estimated effect size (d) following Cohen’s distribution and determine the size of the difference between the two groups [8,9].

**Metabolites identification**

In the case of LC-MS, the annotation was performed by different approaches and tools. Iterative-MS/MS raw data were imported into two different types of software. The first one, the Lipid Annotator software (Agilent Technologies Inc., Santa Clara, CA, USA) was used following a previously reported method [15]. The Lipid Annotator software built a fragmentation-based (MS/MS) library comprising the m/z of all the precursors identified as lipids by the software and their corresponding RT. The Lipid Annotator method parameters were set as follows: ion species [M+H]+, [M+Na]+, and [M+NH4]+ for positive; and [M-H]-, and [M+CH3COOH-H]- for negative ionization mode. Then, for both ionization modes, Q-Score was set as greater than 50 for feature finding, mass deviation less than 20 ppm, fragment score threshold, and total score for identification greater than 30 and 60, respectively. In this annotation process, all available lipids classes were selected. The second one, MS-Dial version 4.70, a freely available software, was used as a complementary tool for annotation following the recommendations included in the tutorial for LC-MS/MS (data-dependent MS/MS) [16]. The MS-Dial method parameters, such as retention time run, MS1 mass range, MS/MS mass range, and accurate mass tolerance or adducts, were fixed and selected based on the acquisition method, the mobile phases, and previously reported findings [15]. All lipids with their MS/MS spectra annotated by Lipid Annotator and/or MS-Dial were manually confirmed by using the Agilent MassHunter (version 10.0), matching the retention time and MS/MS fragmentation. Finally, a manual spectra interpretation (MS1 and MS/MS) was carried out using the CEU Mass Mediator (CMM), which includes information available in different databases such as KEGG [17], HMDB [18], METLIN [19], LIPID MAPS [20], and an in-house library [21,22] to annotate other metabolites detected during data processing but not identified with Lipid Annotator or MS-Dial. Differential Abundance (DA) of the identified lipids was considered when they meet the following criteria: VIP >1, │p(corr)│>0.6, and jackknife from the validated OPLS-DA model and q-value less than 0.05 by Mann-Whitney U test and/or ANCOVA. Furthermore, for those metabolites ionized in positive and negative modes in LC-MS only positive information was kept to reduce redundant information.

**Supplementary Figures:**


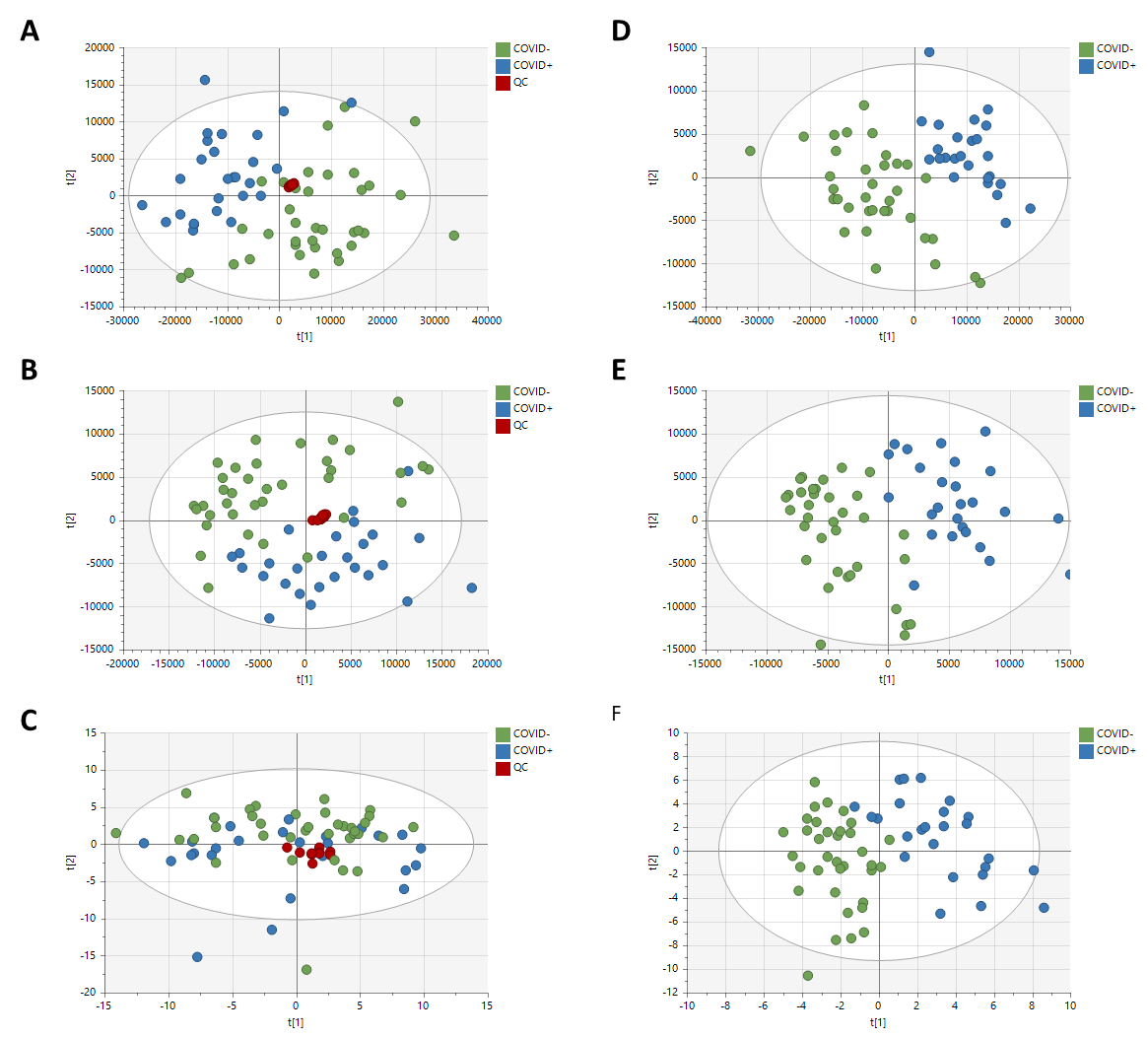


**Figure S1**. **PCA-X and PLS-DA scores plots obtained for COVID-19 positive and COVID-19 negative groups by different metabolomics platforms**. LC-MS data were pareto scaling and GC-MS was autoscaled. In all cases, the samples considered outliers, as well as QCs, were removed and PLS-DA models were built and validated (CV-ANOVA p-value ≤ 0.05). **Plot A – PCA score plot of LC-MS (ESI+)** R^2^ = 0.621; Q^2^ = 0.476. **Plot B –PCA score plot of LC-MS (ESI-)** R^2^ = 0.628; Q^2^ = 0.501. **Plot C –PCA score plot of GC-MS** R^2^ = 0.393; Q^2^ = 0.139. **Plot D – PLS-DA score plot of LC-MS (ESI+)** R^2^ = 0.870; Q^2^ = 0.769; CV-ANOVA = 4.20e-15. **Plot E –PLS-DA score plot of LC-MS (ESI-)** R^2^ = 0.893; Q^2^ = 0.75; CV-ANOVA = 6.83e-15. and **Plot F –PLS-DA score plot of GC-MS** R^2^ = 0.393; Q^2^ = 0.139; CV-ANOVA = 2.95e-10.


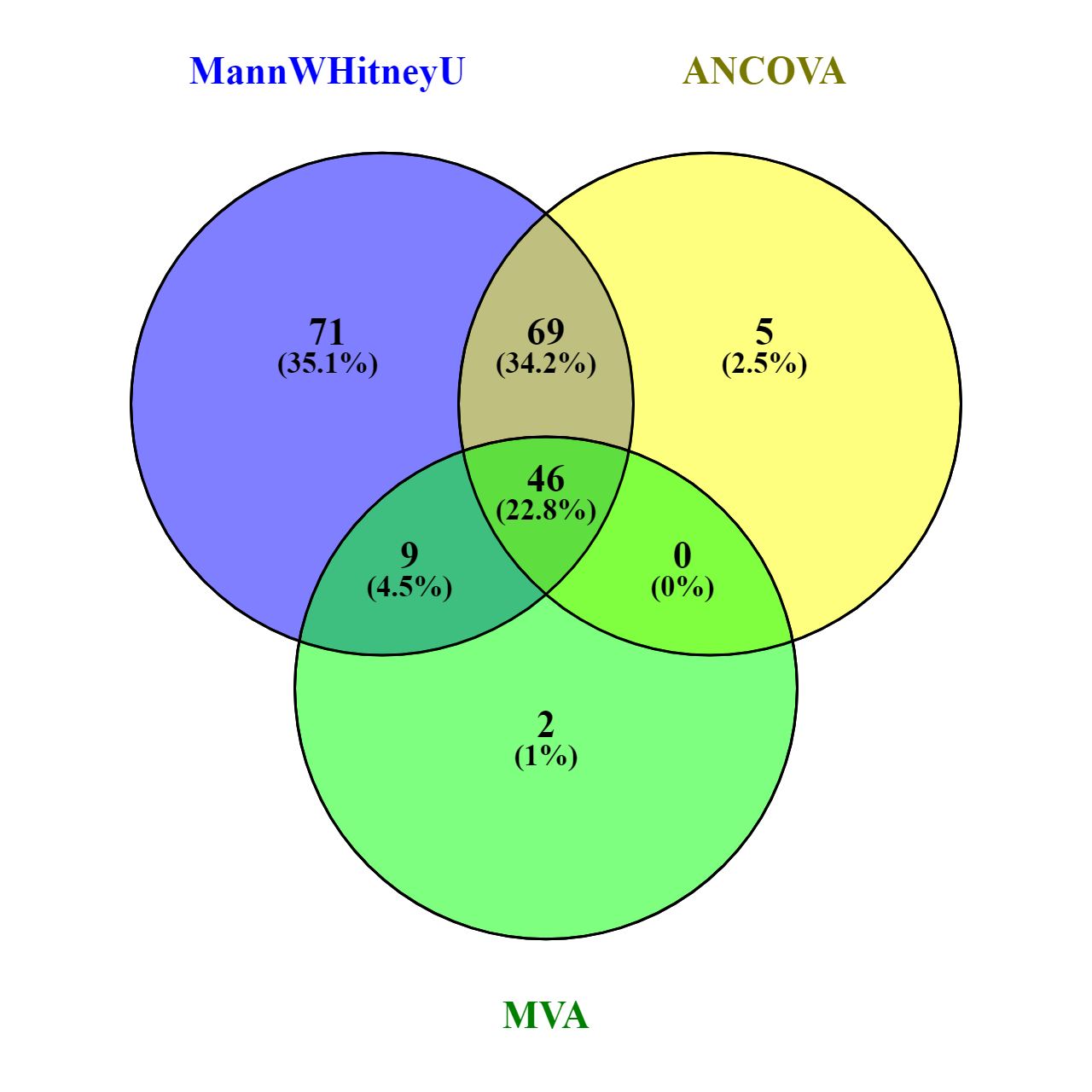


**Figure S2.** **Common significant metabolites obtained by LC-MS and comparing COVID-19+ and COVID-19- groups.** Three statistical analyses are compared: MVA = multivariate analysis, ANCOVA, and MannWhitneyU.


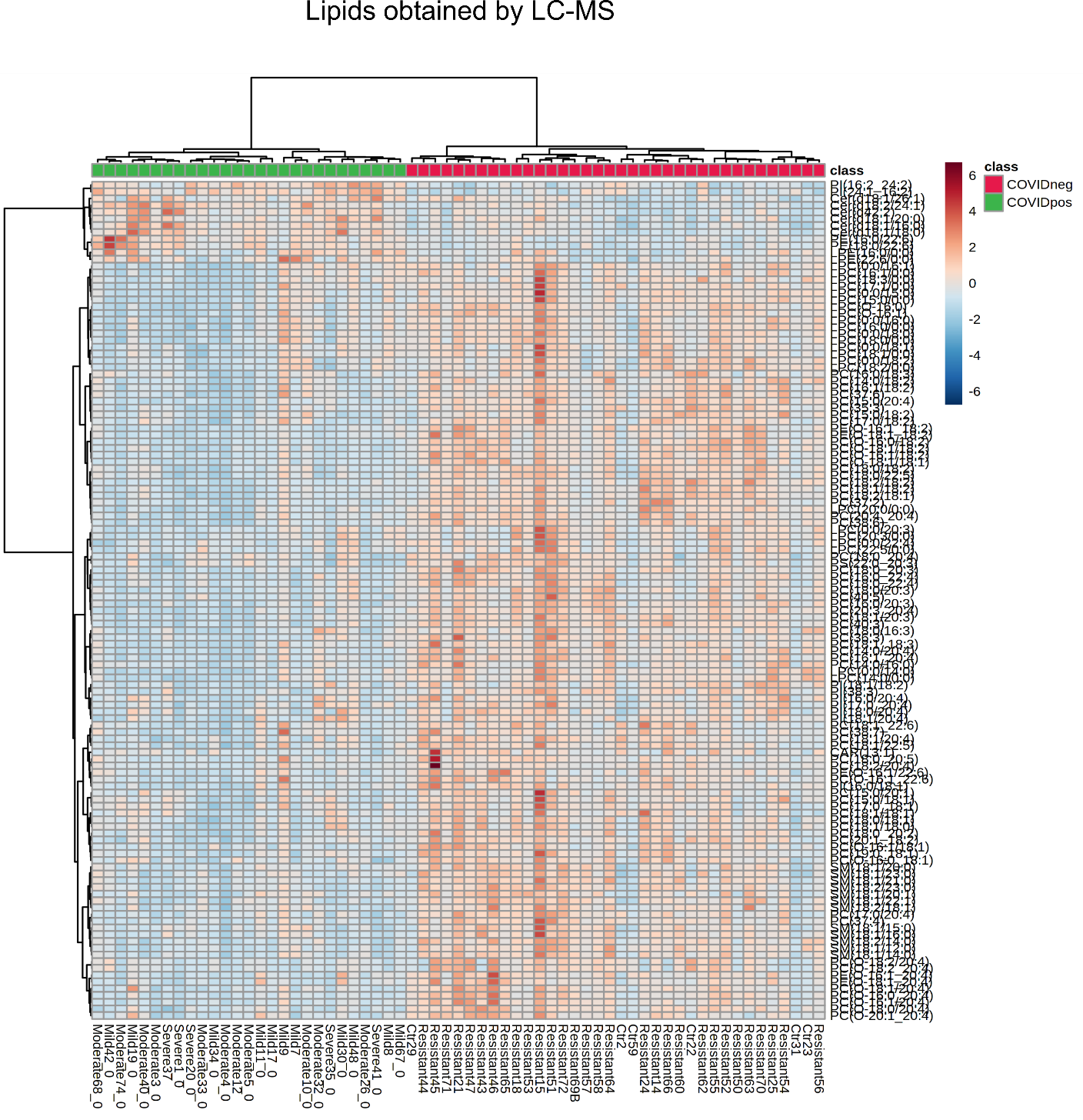


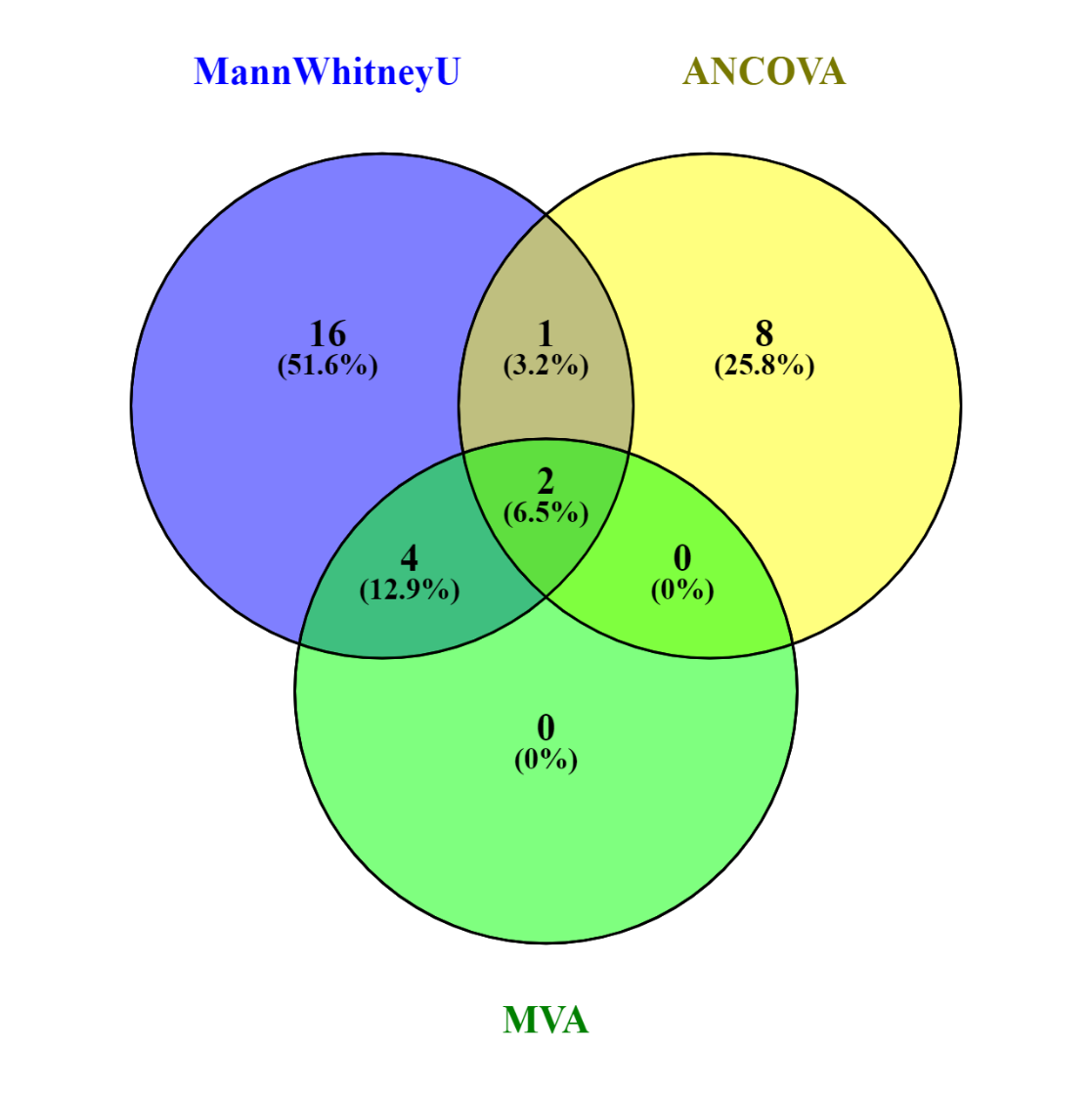


**
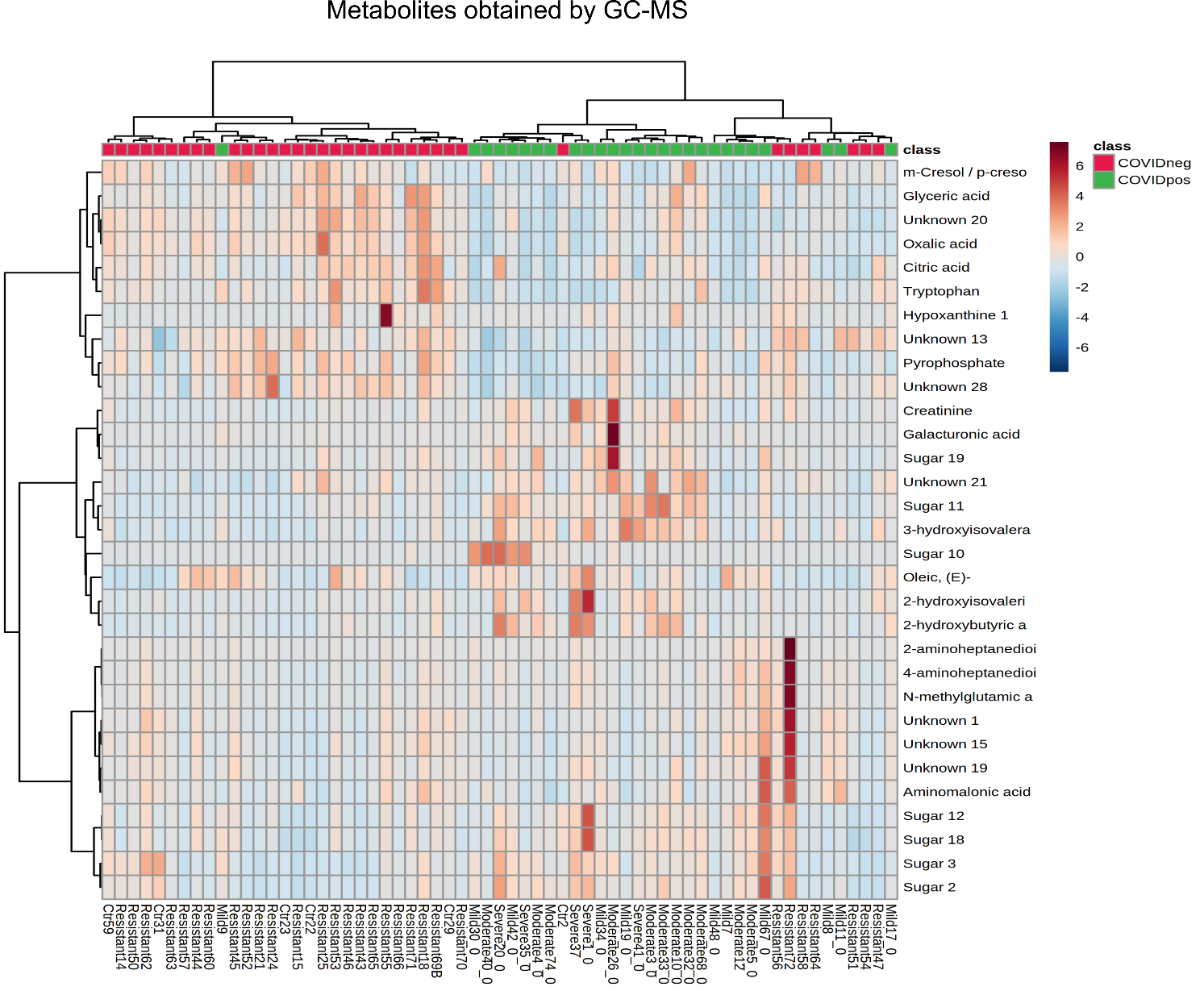
**

**Figure S3. Significant metabolites obtained by GC-MS. Top:** Venn diagram of MannWhitneyU, ANCOVA, and MVA statistical analyses were applied to compare COVID-19+ and COVID-19- groups. **Bottom:** Heatmap of common significant metabolites. The scale color on the right represents the relative abundances. Samples for COVID-19+ patients are depicted in green. Samples for COVID-19- patients are depicted in red. Specific data corresponding to this graph is shown in Table S4-Sheet 2.


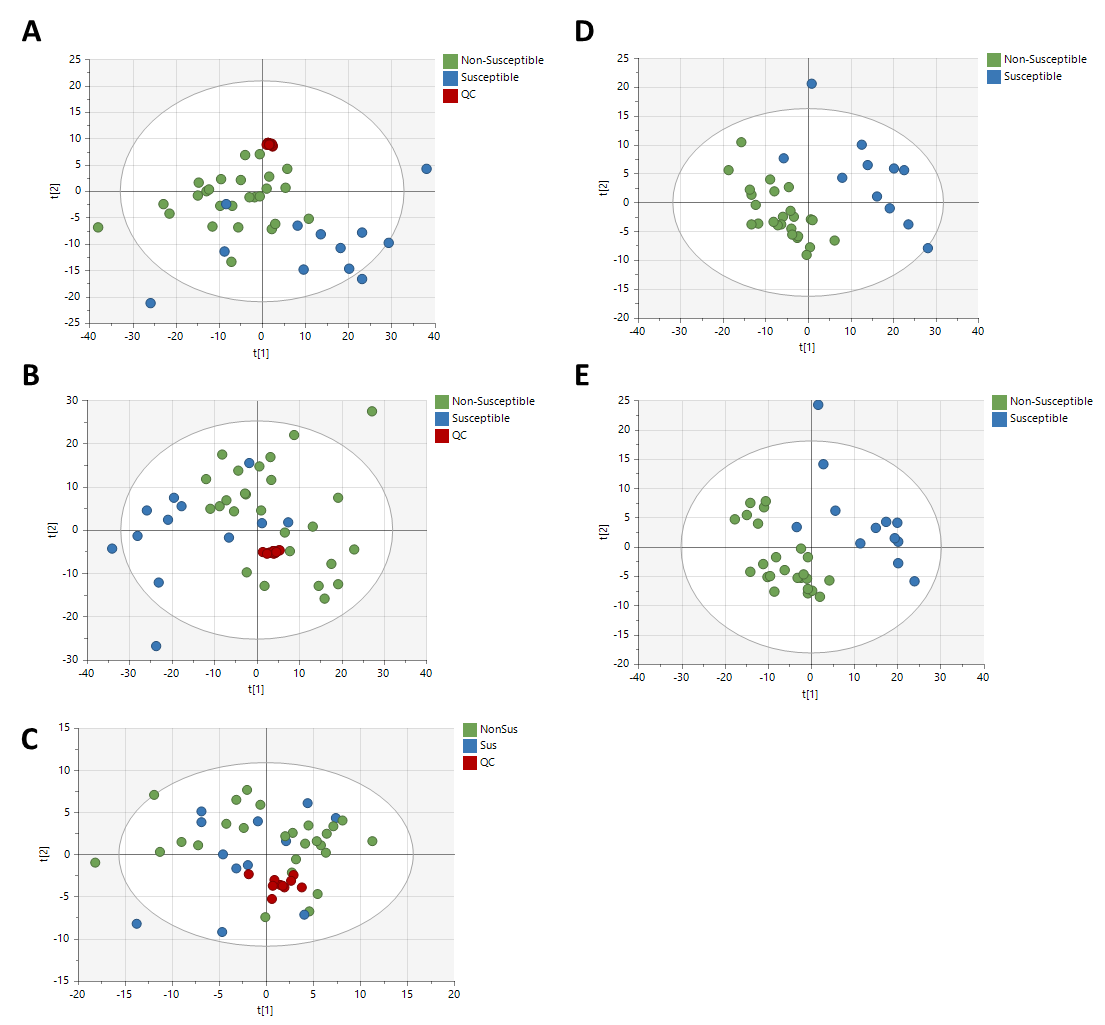


**Figure S4**. **PCA-X and PLS-DA scores plots obtained for susceptible and non-susceptible groups by different metabolomics platforms. Plot A – PCA score plot of LC-MS (ESI+)** R^2^ = 0.493; Q^2^ = 0.340. **Plot B –PCA score plot of LC-MS (ESI-)** R^2^ = 0.470; Q^2^ = 0.347. **Plot C – PCA score plot of GC-MS** R^2^ = 0.493; Q^2^ = 0.340. **Plot D – PLS-DA score plot of LC-MS (ESI+)** R^2^ = 0.861; Q^2^ = 0.699; CV-ANOVA = 1.36e-7. **Plot E –PLS-DA score plot of LC-MS (ESI-)** R^2^ = 0.843; Q^2^ = 0.629; CV-ANOVA = 2.66e-6.

**
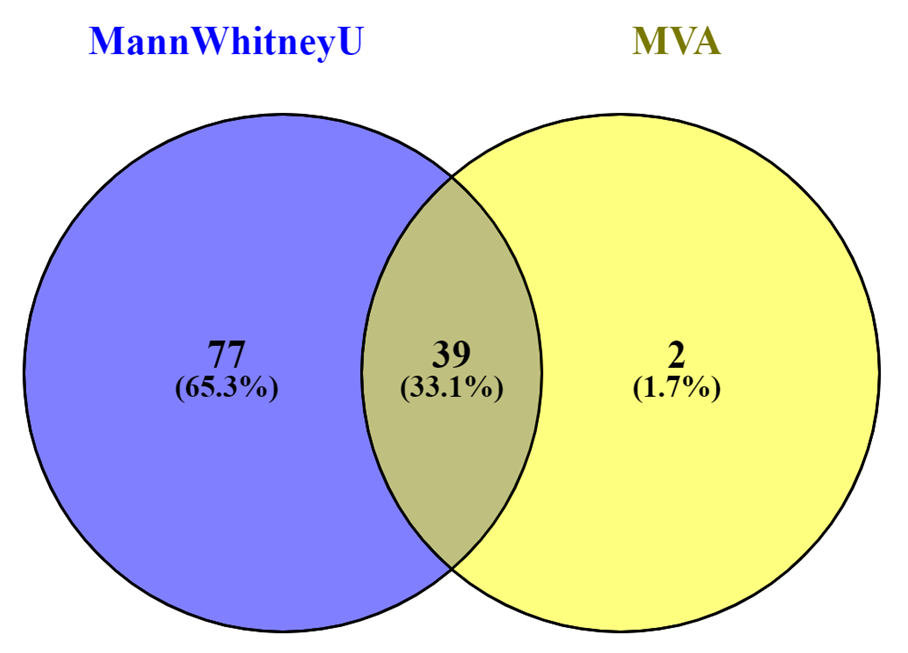
**

**Figure S5.** **Significant metabolites obtained by GC-MS comparing susceptible and non-susceptible groups.** MVA = multivariate analysis. ANCOVA was not applied here because of a similar representation of potential confounding factors. One-third (33.1 %) of significant metabolites were commonly found in multivariate and Mann-Whitney U tests


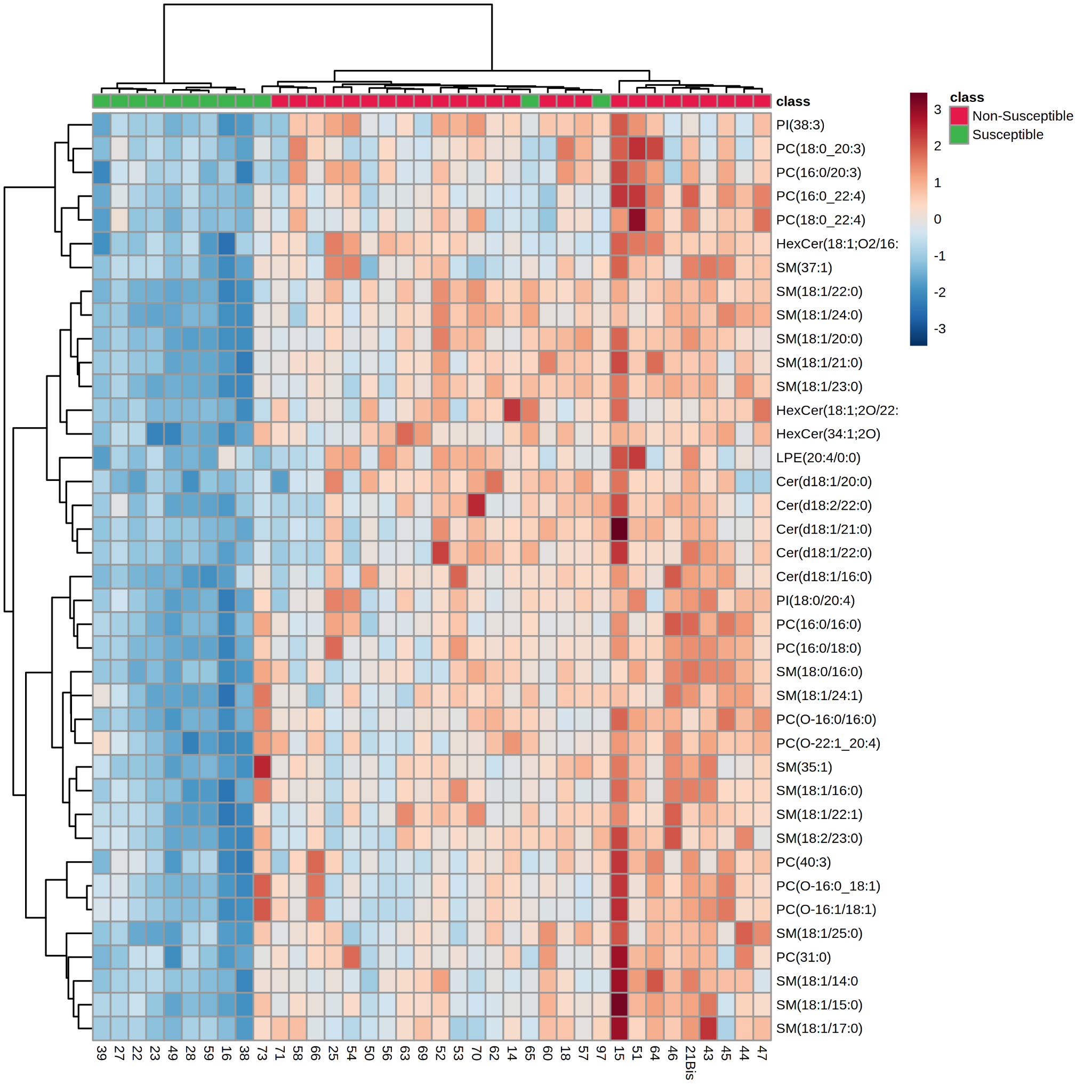


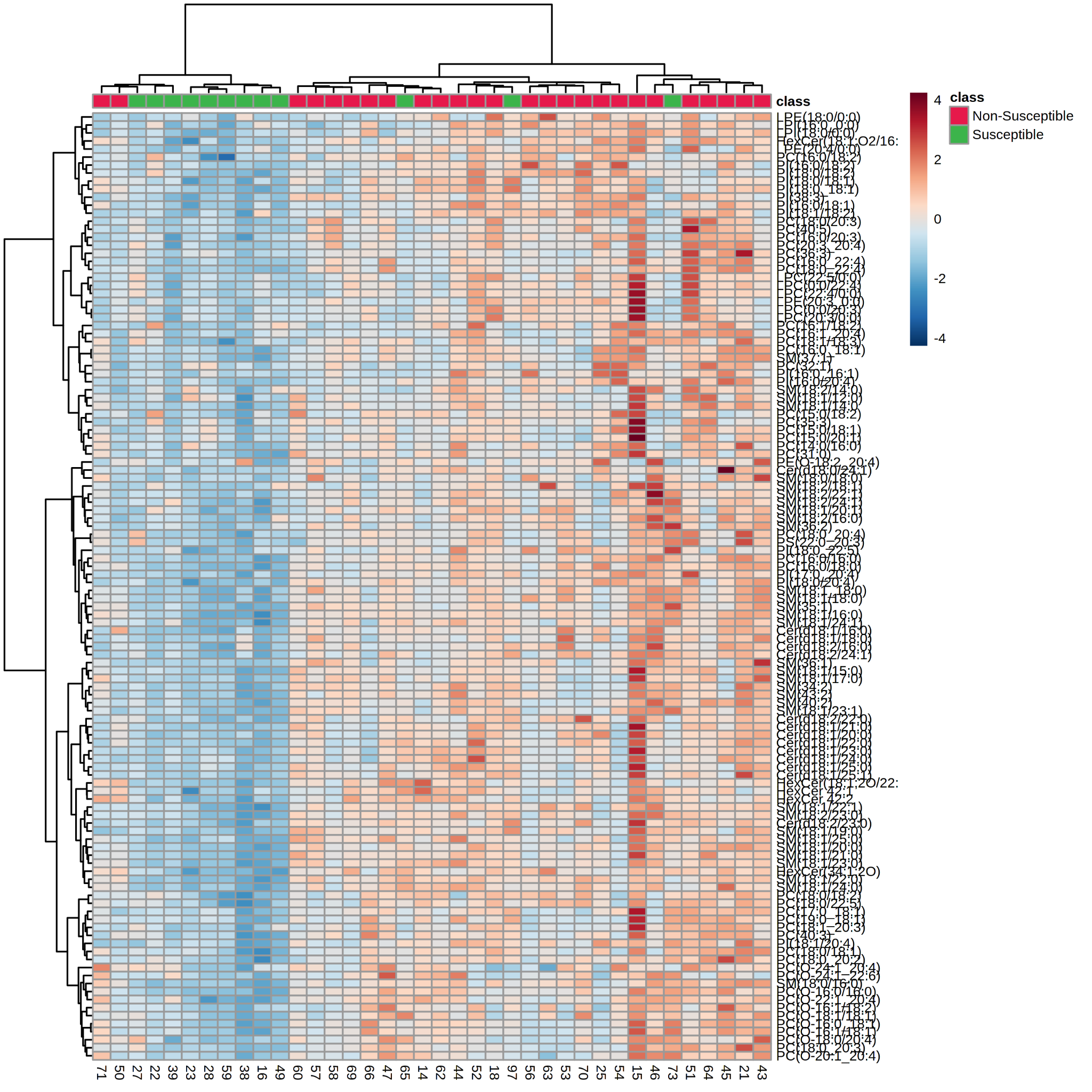


**Figure S6. Heatmaps obtained using the significant metabolites obtained by LC-MS and comparing susceptible and non-susceptible groups.** The scale color on the right represents the relative abundances. Samples for the susceptible group are depicted in green. Samples for the non-susceptible group are depicted in red. Samples names are depicted below and lipid names in the right. Detailed information about this graph can be found in Table S6.Distance measure: Pearson. Clustering method: Ward**. Top:** only for significant metabolites. **Bottom:** heatmap for all metabolites.


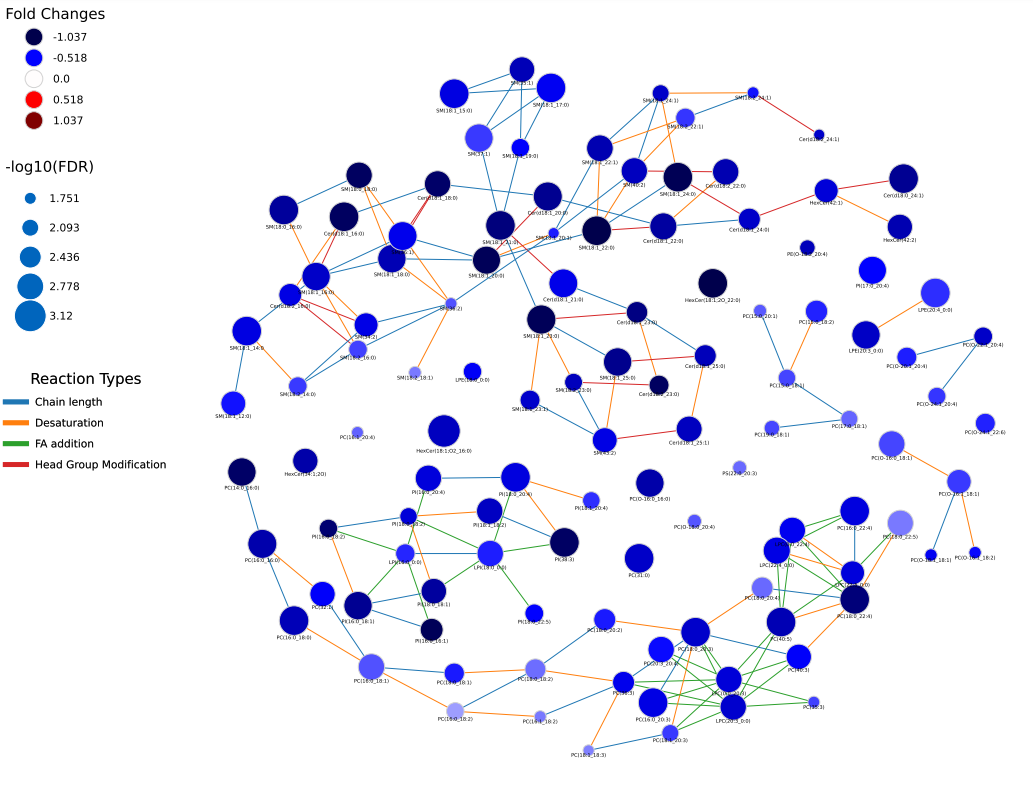


**Figure S7. Lipid network connections generated by LINEX based on data from LC-MS and the comparison of susceptible vs non-susceptible groups.** All significant metabolites obtained by the Mann-Whitney U test and multivariate statistical analysis were introduced in LINEX. Node colors represent the fold change based on susceptible compared to non-susceptible, which means red nodes are upregulated in susceptible and blue nodes are upregulated in non-susceptible. Node size is -log10 (FDR), which is related to q-value, and line colors represent the reaction types that occurred between two metabolites indicated in the legend.

**Supplementary Tables info:**

**Table S1. Total data matrix from all metabolomics techniques used.** Includes the corrected areas of each analytical technique GCMS, LCMSPos, and LCMSNeg together with the sample infection label, group, and sample name.

**Table S2**. **Common metabolites found to be statistically significant from multivariate statistical analysis (MVDA).** It contains two sheets: the first one “COVID+ vs COVID” gathers the significant metabolites obtained for LC-MS and GC-MS by several statistical approaches (Mann Whitney U, ANCOVA, and Multivariate analysis – MVA) when COVID-19+ and COVID-19- individuals are compared. Also, for both analytical techniques, a Venn diagram including this information to see common significant metabolites are included. The second one “Sus vs Non-Sus” includes the same but when susceptible and non-susceptible individuals are considered**.** The selected hits fulfill VIP > 1 and │p (corr)│>0.5 for any of the comparisons performed. Detected signals: 588 at positive and 686 at negative ionization modes. Only the signals with a CV less than 30 % in QCs, a proper peak shape, and an adequate isotopic pattern in both, positive and negative ionization modes, were selected as metabolites. Similarly, 115 metabolites were found in plasma profiles by GC-MS.

**Table S3***.* **Differential metabolites found by LC-MS and significant for COVID-19 disease (COVID-19+ vs COVID-19-).** Contains 5 sheets with the results of significant metabolites obtained by LC-MS when COVID-19+ and COVID-19- individuals are compared. “Mann-Whitney U” gathers the 195 significant lipids obtained by Mann Whitney-U test with the Feature ID, Mass, RT, Specie identified, LINEX Name, Annotation level, q-value, dCohen (effect size), and so on, as well as the areas in each sample. “ANCOVA” includes the 120 significant lipids obtained by the ANCOVA test with the same information as that in the “Mann-Whitney U” sheet. “MVA” contains the 57 significant lipids obtained by OPLS-DA models with VIP values and absolute p(corr) greater than 1 and 0.8, respectively with the information previously mentioned in previous sheets. “LINEX_AllSigMet” includes all the significant lipids obtained by the three approaches (202 lipids in total) that are visually included in Figure S2 together with the percentage of variation colored in red or blue based on the tendency. These colors fit with the colors of the nodes included in Figure S3. Finally, “LINEX_CommonSigMet” similar to “LINEX_AllSigMet” contains only the common significant metabolites obtained by the three statistical approaches (46 lipids) and the tendency of the color fits with Figure 3 node colors to see the upregulated or downregulated tendencies.

**Table S4.** **Metabolites found by GC-MS and significant for COVID-19 disease (COVID-19+ vs COVID-19-).** It has two sheets. The first (“COVID+COVID-_SigMet_Identified”) gathers the significant metabolites, with the tendency, q-value, fold change, percentage of variation, and effect size, obtained by Mann Whitney-U test, ANCOVA, and multivariate statistical analysis when COVID-19+ and COVID-19- individuals are compared. The Venn Diagram shows the common and uncommon metabolites among the three approaches. In short, 2 metabolites are significantly obtained by the three approaches, whereas the remaining 29 metabolites are significant by at least one statistical approach. Specifically, for the comparison of COVID-19+ and COVID-19- groups, 60.87 % and 90.91 % of the significant metabolites obtained by Mann-Whitney U or ANCOVA, respectively, showed effect sizes greater than 0.8. The second (“HeatmapAbunSigMet_COVID+COVID-“) includes the area of each metabolite (31 metabolites in total) for each sample. Correspond to significant metabolites detected by comparison of COVID-19+ and COVID-19- groups analyzed by GC-MS that are depicted as a heatmap in Figure 4.

**Table S5.** **Differential metabolites found by LC-MS and significant when two-by-two comparisons are performed using different disease progression states (mild, moderate, and severe)**. In total, 9 lipids significantly distinguish mild and moderate individuals, 17 mild and severe, and 5 moderate and moderate patients. The “GC-MS” sheet, similar to “LC-MS”, contains 2 significant metabolites that differentiate the three progression groups (mild, moderate, and severe) together with the two-by-two comparisons results. 2 metabolites significantly distinguish mild and moderate individuals, 5 mild and severe, and 9 moderate and moderate patients.

**Table S6.** **Differential metabolites found by LC-MS and significant for susceptible vs non-susceptible comparison.**  Similar to Table S2, this table contains 4 sheets with the results of significant metabolites: “Mann-Whitney U” gathers the 116 significant lipids obtained by Mann Whitney-U test with the Feature ID, Mass, RT, Specie identified, LINEX Name, Annotation level, q-value, dCohen (effect size), and so on, as well as the areas in each sample. “MVA” contains the 41 significant lipids obtained by OPLS-DA models with VIP values and absolute p(corr) greater than 1 and 0.8, respectively. “LINEX_AllSigMet” includes all the significant lipids obtained together with the percentage of variation colored in red or blue based on the tendency. These colors fit with the colors of the nodes included in Figure S9. Finally, “LINEX_CommonSigMet” similar to “LINEX_AllSigMet” contains only the common significant metabolites obtained by the three statistical approaches (38 lipids) and the tendency of the color fits with Figure 8 node colors to see the upregulated or downregulated tendencies.

**References for Supplementary Information:**

1. Liakh I, Sledzinski T, Kaska L, Mozolewska P, Mika A. Sample Preparation Methods for Lipidomics Approaches Used in Studies of Obesity. Molecules. **2020**; 25(22):5307.

2. Züllig T, Trötzmüller M, Köfeler HC. Lipidomics from sample preparation to data analysis: a primer. Anal Bioanal Chem. **2020**; 412(10):2191–2209.

3. Gonzalez-Riano C, Gradillas A, Barbas C. Exploiting the formation of adducts in mobile phases with ammonium fluoride for the enhancement of annotation in liquid chromatography-high resolution mass spectrometry based lipidomics. J Chromatogr Open. **2021**; 1:100018.

4. Garcia A, Barbas C. Gas chromatography-mass spectrometry (GC-MS)-based metabolomics. Methods Mol Biol Clifton NJ. **2011**; 708:191–204.

5. Naz S, Garcia A, Rusak M, Barbas C. Method development and validation for rat serum fingerprinting with CE-MS: application to ventilator-induced-lung-injury study. Anal Bioanal Chem. **2013**; 405(14):4849–4858.

6. Kuligowski J, Sánchez-Illana Á, Sanjuán-Herráez D, Vento M, Quintás G. Intra-batch effect correction in liquid chromatography-mass spectrometry using quality control samples and support vector regression (QC-SVRC). The Analyst. **2015**; 140(22):7810–7817.

7. Godzien J, Ciborowski M, Angulo S, Barbas C. From numbers to a biological sense: How the strategy chosen for metabolomics data treatment may affect final results. A practical example based on urine fingerprints obtained by LC-MS. Electrophoresis. **2013**; 34(19):2812–2826.

8. Smilde AK, Jansen JJ, Hoefsloot HCJ, Lamers R-JAN, Greef J van der, Timmerman ME. ANOVA-simultaneous component analysis (ASCA): a new tool for analyzing designed metabolomics data. Bioinforma Oxf Engl. **2005**; 21(13):3043–3048.

9. Khammar A, Yarahmadi M, Madadizadeh F. What Is Analysis of Covariance (ANCOVA) and How to Correctly Report Its Results in Medical Research? Iran J Public Health. **2020**; 49(5):1016–1017.
